# Autophagy-mediated ID1 turnover dictates chemo-resistant fate in ovarian cancer stem cells

**DOI:** 10.1186/s13046-024-03147-z

**Published:** 2024-08-10

**Authors:** Pratham Phadte, Aniketh Bishnu, Pranay Dey, Manikandan M, Megha Mehrotra, Prerna Singh, Shritama Chakrabarty, Rounak Majumdar, Bharat Rekhi, Malay Patra, Abhijit De, Pritha Ray

**Affiliations:** 1 Imaging Cell Signalling & Therapeutics Lab, Advanced Centre for Treatment, Research and Education in Cancer, Tata Memorial Centre, Navi Mumbai, 410210 India; 2 Molecular Functional Imaging Lab, Advanced Centre for Treatment, Research and Education in Cancer, Tata Memorial Centre, Navi Mumbai, 410210 India; 3 Homi Bhabha National Institute, Anushakti Nagar, Mumbai, 400094 India; 4 Laboratory of Medicinal Chemistry and Cell Biology, Department of Chemical Sciences, Tata Institute of Fundamental Research, Mumbai, 400005 India; 5 Department of Pathology, Tata Memorial Hospital, Mumbai, 400012 India; 6 Indian Institute of Science Education and Research, Bhopal, 462066 India; 7 Indian Institute of Science Education and Research, Kolkata, 741246 India

**Keywords:** Autophagy, Cancer stem cells, Chemo-resistance, Differentiation, Epithelial Ovarian Cancer, ID1, SLC31A1, TCF12

## Abstract

**Background:**

The mechanisms enabling dynamic shifts between drug-resistant and drug-sensitive states in cancer cells are still underexplored. This study investigated the role of targeted autophagic protein degradation in regulating ovarian cancer stem cell (CSC) fate decisions and chemo-resistance.

**Methods:**

Autophagy levels were compared between CSC-enriched side population (SP) and non-SP cells (NSP) in multiple ovarian cancer cell lines using immunoblotting, immunofluorescence, and transmission electron microscopy. The impact of autophagy modulation on CSC markers and differentiation was assessed by flow cytometry, immunoblotting and qRT-PCR. In silico modeling and co-immunoprecipitation identified ID1 interacting proteins. Pharmacological and genetic approaches along with Annexin-PI assay, ChIP assay, western blotting, qRT-PCR and ICP-MS were used to evaluate effects on cisplatin sensitivity, apoptosis, SLC31A1 expression, promoter binding, and intracellular platinum accumulation in ID1 depleted backdrop. Patient-derived tumor spheroids were analyzed for autophagy and SLC31A1 levels.

**Results:**

Ovarian CSCs exhibited increased basal autophagy compared to non-CSCs. Further autophagy stimulation by serum-starvation and chemical modes triggered proteolysis of the stemness regulator ID1, driving the differentiation of chemo-resistant CSCs into chemo-sensitive non-CSCs. In silico modeling predicted TCF12 as a potent ID1 interactor, which was validated by co-immunoprecipitation. ID1 depletion freed TCF12 to transactivate the cisplatin influx transporter SLC31A1, increasing intracellular cisplatin levels and cytotoxicity. Patient-derived tumor spheroids exhibited a functional association between autophagy, ID1, SLC31A1, and platinum sensitivity.

**Conclusions:**

This study reveals a novel autophagy-ID1-TCF12-SLC31A1 axis where targeted autophagic degradation of ID1 enables rapid remodeling of CSCs to reverse chemo-resistance. Modulating this pathway could counter drug resistance in ovarian cancer.

**Supplementary Information:**

The online version contains supplementary material available at 10.1186/s13046-024-03147-z.

## Background

Cancer Stem Cells (CSCs) are a subset of cancer cells with stem-like properties underlying tumor propagation, metastasis, and therapeutic failure [1]. By exploiting their drug resistance properties, CSCs can be functionally isolated by Side population (SP) assay where SP cells, by virtue of elevated ATP-binding cassette (ABC) transporters, efflux DNA binding dyes like “Dye Cycle Voilet” (DCV) or Hoechst 33342 [2]. Superior effluxing abilities enable SP cells to evade chemotherapeutics, causing treatment resistance and relapse [3]. However, how these CSCs maintain their superior resistance fate in a comparatively homogenous pool of cancer cells (cell lines) or a heterogenous tumor cell population is yet to be clarified.

Autophagy is emerging as a key pathway controlling cell fate decisions [4]. CSCs from various tumor types exhibit increased/complete basal autophagy that helps to maintain the primary stem-like properties which further gets modulated with elevation of autophagy due to intra-tumoral stresses [5]. However, the direct molecular effectors and signaling pathways connecting different functional features of CSCs, especially for mechanisms linking autophagy to drug resistance phenotypes, are less defined. Such knowledge is virtually unavailable for CSC-enriched tumor spheroids from patients with recurrent disease. Identifying specific autophagy substrates and delineating pathways by which targeted autophagic turnover directs CSC fate and drug response could reveal vulnerabilities to developing more efficient therapeutic strategies for eliminating this resistant pool.

An emerging family of transcriptional repressors that majorly control the timing of cell fate determination and differentiation in embryonic stem cells, adult stem cells and progenitor cells during normal development and adult life are the Inhibitor of DNA binding (ID) proteins [6]. These proteins are frequently deregulated in many cancers [7], including ovarian cancer [8, 9], and they endow cancer cells with biological features similar to normal stem cells [10]. The members of these basic helix-loop-helix (bHLH) transcription factors lack a basic DNA binding domain and inhibit the transcriptional activities of other bHLH transcription factors through dominant-negative hetero-dimerization, which are critical for promoting differentiation, organogenesis, senescence [11]. While numerous studies have demonstrated the effects of ID1 on promoting self-renewal in colon CSCs [12], inhibiting apoptosis in ovarian cancer cells [13], and mediating chemo-resistance in nasopharyngeal carcinoma cells [14], glioblastoma stem cells [15], ovarian cancer cells [13, 16], esophageal squamous cell carcinoma cells [17], gastric cancer cells [18], and colorectal cancer cells [19], the majority of these studies have relied on indirect evidence from knockdown/knockout or overexpression experiments. These studies have shown that manipulating ID1 levels can influence the expression of genes or pathways involved in self-renewal, apoptosis, and drug resistance pathways. However, the precise mechanism and direct interacting partners of ID1 involved in CSC differentiation and their drug response are yet to be identified. To date, only two studies have identified physical interaction of ID1 with either p65 or cMYC and demonstrated their contributions towards activation of NF-kB signaling pathway [20] or pentose phosphate pathway with an emphasis on chemoresistance [21]. However, a direct connection between ID1 modulation with drug influx/efflux pumps and platinum accumulation were not shown. Thus, a comprehensive picture of the ID1-regulated molecular players and the mechanism involved in the differentiation and drug response of the CSCs is warranted.

Herein, we explored whether dynamic autophagic control over ID1 levels could elicit rapid shifts in cell fate and drug response in the CSC-enriched population. Our data demonstrates that autophagic degradation of the ID1 enables rapid differentiation of SP cells into NSP cells, leading to enhanced platinum sensitivity in both acquired and intrinsically platinum-resistant epithelial ovarian cancer cells. In silico modeling predicted the ID1 interactor TCF12 as a candidate downstream effector. Using genetic, pharmacological, and autophagy-mediated approaches for ID1 downregulation, we, for the first time, showed that TCF12, when freed from the repressive clutches of ID1, transactivated the cisplatin influx transporter SLC31A1, thereby reprogramming the SP cells from an efflux-high, chemo-resistant state to an influx-high, chemo-sensitive state. Such reprogramming led to higher uptake of cisplatin. The association between elevated autophagy, ID1 and SLC31A1 expression was also observed in the CSC-enriched spheroids of relapsed EOC patients who responded again to platinum-based drugs. Altogether, our work, for the first time, reveals that autophagic degradation of key regulatory proteins exerts rapid control over ovarian CSC fate decisions to reverse drug resistance.

## Methods

### Cell lines and patient-derived tumor cell models and culture conditions

Acquired chemoresistance cell model: The A2780^DualLR^ cell line was developed through incremental does of platinum-taxol over 6 months, and it displays high resistance to cisplatin (ten times higher than the IC50 of parental A2780 cells). It was developed through incremental dose of platinum selection with platinum-taxol over 6 months to model acquired cisplatin resistance [22]. Intrinsic chemoresistance cell model: TOV21G cells have previously been shown to exhibit intrinsic cisplatin resistance which was established from a primary malignant adenocarcinoma of a 62-year old women (ATCC, catalog number CRL-11730) [23]. A2780^DualLR^ and TOV21G were cultured in DMEM (GIBCO, 12,800,017, Thermo Fisher Scientific, Waltham, MA, USA) and RPMI (GIBCO, 11,875,093, Thermo Fisher Scientific, Waltham, MA, USA), respectively, supplemented with 10% fetal bovine serum (HIMEDIA, RM10409, HiMedia Laboratories, Mumbai, India) and 1% penicillin–streptomycin (HIMEDIA, A002, HiMedia Laboratories, Mumbai, India). Cells were maintained at 37 °C in a humidified 5% CO2 atmosphere.

Patient-derived tumor cell models: Though primary treatment regime for EOC consists of platinum and taxol drugs, platinum-resistance and relapse are the major hinderance to successful treatment. Clinically recurrent patients are grouped into platinum resistant relapse patients (recurrence occur within 6-months of the last treatment and who never respond to second platinum treatment after relapse) and platinum-sensitive relapse patients (recurrence occur beyond 6-months of the last treatment and who again respond to re-challenge with platinum after relapse). Accrual of patients were done as per approval from the Institutional Ethics Committee-III at ACTREC-TMC. Written informed consent was obtained from all patients prior to inclusion in the study. The malignant ascitic fluid was collected from chemo-naïve (treatment was not started) (n = 6), platinum-sensitive relapse (n = 5) and platinum-resistant relapse (n = 3) high-grade serous ovarian cancer patients, and the floating spheroids population are collected after low speed centrifugation to separate them from suspended single cells. All the experiments were performed within one week of obtaining the spheroids.

### Treatments

Cells were treated with 500 nM Torin1 (Calbiochem, 475,991) to induce autophagy. Stock concentration of 10 mM Torin 1 was made in DMSO, diluted 1:100 in PBS, and 5 µL of this solution was added to 1 mL media to achieve a final concentration of 500 nM. Autophagy inhibition was achieved by incubating cells in complete media with 10 μM chloroquine (CQ) (Sigma-Aldrich, C6628) for 24 h. A 10 mM stock was made in PBS and diluted 1:1000 in culture media. For ID1 rescue experiments, cells were treated with 100 ng/mL bortezomib (Sigma-Aldrich, 5.04314) made in PBS and 10 μM CQ for 24 h. The pan-ID protein antagonist AGX51 (MedChemExpress, HY-129241) was used at a final concentration of 40 μM. A 10 mM stock solution was made in DMSO and diluted 1:250 in media. Vehicle controls using equivalent volumes of DMSO was included where ever appropriate.

### Side population assay

Side population and non-side population cells were sorted using the Dye Cycle Violet (DCV) dye exclusion assay [2, 24] (Invitrogen, V35003, Thermo Fisher Scientific, Waltham, MA, USA). Sorting was performed on a BD FACS Aria-I fitted with a violet laser, as previously described [25]. The membrane drug transporter inhibitor Verapamil (50 μM) was utilized as a negative control to validate SP gating. Data analysis was done using FLOWJO software.

### Immunoblotting

Cell lysates were prepared in RIPA buffer (50 mM Tris pH 7.4, 150 mM NaCl, 1% NP-40, 0.5% sodium deoxycholate, 0.1% SDS) with protease and phosphatase inhibitors (Sigma-Aldrich, PPC1010, Merck KGaA, Darmstadt, Germany) and protein concentration determined by Bradford assay (Sigma-Aldrich, B6916, Merck KGaA, Darmstadt, Germany). Based on the linear range of detection, 30–60 μg of protein were separated on 8–12% polyacrylamide gels and transferred to PVDF membranes (PALL, 741,260, Pall Corporation, Port Washington, NY, USA). Membranes were blocked with 5% BSA in TBS with 0.05% Tween 20 (Sigma-Aldrich, P1379, Merck KGaA, Darmstadt, Germany) for 1 h, followed by overnight incubation at 4 °C with primary antibodies. After washing three times in TBST, membranes were incubated for 2 h at room temperature with HRP-conjugated respective secondary antibodies. Blots were developed using a chemiluminescent substrate (Takara, T7101A, Takara Bio Inc., Kusatsu, Shiga, Japan) and imaged on a GelDoc system (Bio-Rad). Quantification was performed using ImageJ software (NIH). All immunoblotting experiments were performed with a minimum of three biological replicates (n ≥ 3) unless otherwise stated.

### Confocal microscopy

For confocal microscopy, cells and patient-derived spheroids were fixed with 4% paraformaldehyde and permeabilized with 0.2% Triton X-100 (for nuclear proteins), followed by blocking with 3% BSA in PBS for 30 min at room temperature. Samples were incubated overnight at 4 °C with primary antibodies, washed three times in PBS, and then incubated for 2 h at room temperature with secondary antibodies. After further washing in PBS, samples were mounted on glass slides using Vectashield Antifade Mounting Medium (Vector Labs, H-1000–10, Vector Laboratories, Burlingame, CA, USA). Imaging was performed on a Carl Zeiss LSM 780 confocal microscope using a 63 × objective with 488, 568, and 633 lasers. Quantification of LC3, ID1, TCF12, LC3/LAMP1, LC3/ID1 and LC3/TCF12 puncta and mean fluorescence values was done using Zen and ImageJ software. Primary and secondary antibodies utilized are listed in Table S1. A minimum of 50 cells were analyzed per condition across at least 5 random fields of view. Experiments were performed in at least two biological replicates. Isotype controls (rabbit IgG for TCF12 & LC3; mouse IgG for ID1) were performed to confirm antibody specificity.

### Transmission electron microscopy

Cell pellets were fixed using 2.5% glutaraldehyde (volume: volume) in 0.1 M cacodylate buffer (pH 7.4) followed by post-fixation for 1 h at 4 °C with 1% osmium tetroxide (weight: volume) in 0.1 M cacodylate buffer. Samples underwent serial dehydration in graded ethanol. Embedding was performed in Araldite resin with polymerization at 70 °C for 24 h. Using a Leica UC7 ultramicrotome, ultrathin Sects. (50-70 nm) were cut from the blocks and mounted on 300 mesh copper grids. Contrast enhancement was done by staining with 10% uranyl acetate in alcohol and lead citrate. Imaging was performed on a JEOL 1400 plus transmission electron microscope operated at 120 kV using iTEM software and the Tengra camera. At least 12 cells were analyzed per condition across multiple fields with n = 2 replicates.

### Cell viability assay

Chemo-resistant A2780^DualLR^ (1200 cells/well) and TOV21G (1000 cells/well) cells were treated with varying concentrations of cisplatin for 72 h. Cell viability was determined by the standard MTT (Sigma-Aldrich M2128, Merck KGaA, Darmstadt, Germany) assay. The formula Absorbance [(Test)/Absorbance (Control) × 100] was used to calculate viability. Cells were incubated with 10% MTT solution for 2 h, followed by the addition of 100 μL DMSO. Absorbance was measured at 570 nm with background correction at 630 nm. All cell viability assays were performed in triplicate, with three independent biological replicates (n = 3).

### Lentiviral-mediated sh-RNA constructs for PAN-ID and ID1 silencing

Lentiviral knockdown constructs were generated in the pLL3.7 vector using target sequences provided in Table S2. Lentiviral particles were produced as previously described [26] by transfecting 293FT cells with the lentiviral vector plasmid, P-delta packaging plasmid, and VSVG envelope plasmid at a 4:2:1 ratio. At 60 h post-transfection, lentiviruses were collected and used to transduce A2780^DualLR^ and TOV21G cells. Cells stably expressing the shRNA constructs were enriched by fluorescence-activated cell sorting based on EGFP expression. Scrambled shRNA controls (scrPAN-ID and scrID1) were used to account for non-specific effects of shRNA expression.

### Apoptosis detection with Annexin/PI

Cells were treated with cisplatin (Sigma-Aldrich, P4394, Merck KGaA, Darmstadt, Germany) at 10 × the IC50 concentration for each respective cell line for 24 h. To detect apoptosis, SP, NSP, TP, scrPAN-ID, scrID1, shPAN-ID, and shID1 cells of A2780^DualLR^ and TOV21G cells were stained using the Dead Cell Apoptosis Kit (Invitrogen, V13242 and A35122 Thermo Fisher Scientific, Waltham, MA, USA) following the manufacturer's protocol. In brief, cells were harvested in ice-cold PBS and resuspended in 100 μL of Annexin V binding buffer. 5 μL of Annexin V and 5 μL of propidium iodide (PI) were added and incubated for 15 min in the dark at room temperature. Then, 400 μL of Annexin V binding buffer was added to each tube before analysis of 50,000 events by flow cytometry using the Attune NxT Flow Cytometer (ThermoFisher). Flow cytometry data were analyzed using FlowJo software version 10. Flow cytometry analysis was performed on at least 50,000 events per sample, with three independent biological replicates (n = 3).

### Quantitative real-time PCR

Complementary DNA (cDNA) was synthesized from extracted RNA using the SuperScript IV First-Strand Synthesis kit (Invitrogen, 18,090,010, Thermo Fisher Scientific, Waltham, MA, USA) following the manufacturer's protocol. Quantitative real-time PCR was performed with the PowerUp SYBR Green Master Mix (Invitrogen, A25741, Thermo Fisher Scientific, Waltham, MA, USA) using gene-specific primers, with Glyceraldehyde-3-phosphate-dehydrogenase (GAPDH) as an internal control. Relative gene expression was measured as delta Ct values. The primer sequences utilized can be found in Table S2. All qRT-PCR experiments were performed in technical triplicate, with three independent biological replicates (n = 3).

### In-silico analysis

String (https://string-db.org/) and Agile Network (http://cicblade.dep.usal.es:8080/APID/) analysis was performed to identify the protein–protein interaction network of ID1. The protein sequence of ID1 (155 aa, UniProtKB accession number: P41134) and TCF12 (682 aa, UniProtKB accession number: Q99081) was retrieved from UniProt (http://www.uniprot.org). HADDOCK 2.4 was used to generate 3D structures of ID1-TCF12, which were then fed into 3VEE for Interface Identification, PDBePISA, and PDBsum for Interface residue interaction analysis.

### Co-immunoprecipitation assay (Co-IP)

Cell lysates were prepared using EBC lysis buffer (120 mM NaCl; 0.5% (v/v) Non-idet P-40; 5 μg/mL leupeptin; 10 μg/mL aprotinin; 50 μg/mL PMSF; 0.2 mM sodium orthovanadate; 100 mM NaF; 50 mM Tris–Cl; pH 8.0). 2 mg of cell lysate were incubated overnight at 4 °C with 5 μg of anti-ID1 and anti-TCF12 antibody in EBC lysis buffer, followed by the addition of Dynabeads™-Protein G (Invitrogen, 10003D, Thermo Fisher Scientific, Waltham, MA, USA). Immune complexes were magnetically isolated and washed three times with EBC lysis buffer, then eluted using 2X Laemmli buffer. The presence of ID1 and TCF12 in the co-immunoprecipitation complexes was detected by western blot. A list of primary and secondary antibodies utilized can be found in Table S1. IgG isotype control was included to confirm specific antibody binding. Experiments were performed in duplicate (n = 2).

### Chromatin immunoprecipitation (ChIP)assay

Chromatin immunoprecipitation (ChIP) was carried out as previously described [27] with some modifications. In brief, 25 μg of sonicated DNA was immunoprecipitated using 3 μg of ChIP grade antibody specific for TCF12 (see Table S1). Protein/DNA complexes were collected via magnetic pulldown and eluted from the beads using an elution buffer. The enriched DNA fragments were analyzed by RT-PCR using specific primers (listed in Table S2). Non-immunoprecipitated chromatin served as the input control. All ChIP primers utilized in this study are provided in Table S2. ChIP assays were performed in duplicate (n = 2).

### Platinum quantification using Inductively Coupled Plasma Mass Spectrometry (ICP-MS)

Treatment of cisplatin (Sigma-Aldrich, P4394, Merck KGaA, Darmstadt, Germany) (10 × IC50 values of respective cell line) was given for 8 h. One of the replicates was used to quantify the protein content. The rest of the petri dishes were digested using ICP-MS grade 70% HNO_3_ (400 µL per petri dish, 2 days at room temperature) followed by H_2_O_2_ (400 µL per petri dish, 1 day at room temperature). Samples were then collected in 15 mL tubes, diluted using double distilled water, and metal contents were analyzed by ICP-MS to obtain the whole-cell platinum uptake, which was then normalized to the protein content. ICP-MS experiments were performed in triplicate (n = 3).

### Cell block preparation using the thromboplastin-plasma method

The ascitic fluid sample collected from different group of patients as mentioned above was centrifuged to sediment the cells. The supernatant was removed, and the cell pellet was resuspended in a small amount of plasma of the blood. Thromboplastin reagent was added to the plasma-cell mixture and mixed well. It was allowed to clot for 5 min. The clot was transferred onto a piece of filter paper, wrapped, and placed in a tissue cassette. The tissue cassette was fixed in 10% buffered formalin for at least 4 h. The fixed sample was processed using standard histological techniques to embed in paraffin. Sections of the paraffin block were cut and stained as desired for microscopic examination.

### Immunohistochemistry

Immunohistochemical staining was performed utilizing a commercially available kit (Abcam, ab2364660). In brief, 5 μM sections were cut from cell blocks prepared from primary patient cells. The sections were deparaffinized, hydrated, and treated with peroxide, and antigen retrieval was carried out using sodium citrate buffer (pH 6) heated to 120 °C for 10 min in a pressure cooker. The sections were then incubated with a protein-blocking agent, followed by overnight incubation at 4 °C with an anti-SLC31A1(1:100 dilution) and ID1 antibody (1:50 dilution). Secondary antibody treatment and color development using HRP-conjugated DAB substrate followed. An experienced pathologist, blinded to sample histopathology and patient information, independently reviewed and scored the immunostaining of the formalin-fixed, paraffin-embedded sections.

### Statistical analysis

All statistical analyses were performed using GraphPad Prism 9.0 software (GraphPad Software, San Diego, CA, USA). Data normality was assessed using the Shapiro–Wilk test. For normally distributed data, Student's t-test or Welch's t-test (for unequal variances) was used for two-group comparisons. For multiple group comparisons, one-way ANOVA followed by Dunnett's post-hoc test (for comparisons to a single control group) or Bonferroni correction (for multiple pairwise comparisons) was used. All experiments were performed with a minimum of three biological replicates (n ≥ 3) unless otherwise stated. Data are presented as mean ± standard error of the mean (SEM). Statistical significance was set at *p* ≤ 0.05 with *p < 0.05, **p < 0.01, and ***p < 0.001 indicated in figures.

## Results

### Ovarian cancer stem cells exhibit a high basal autophagy

**Fig. 1:**
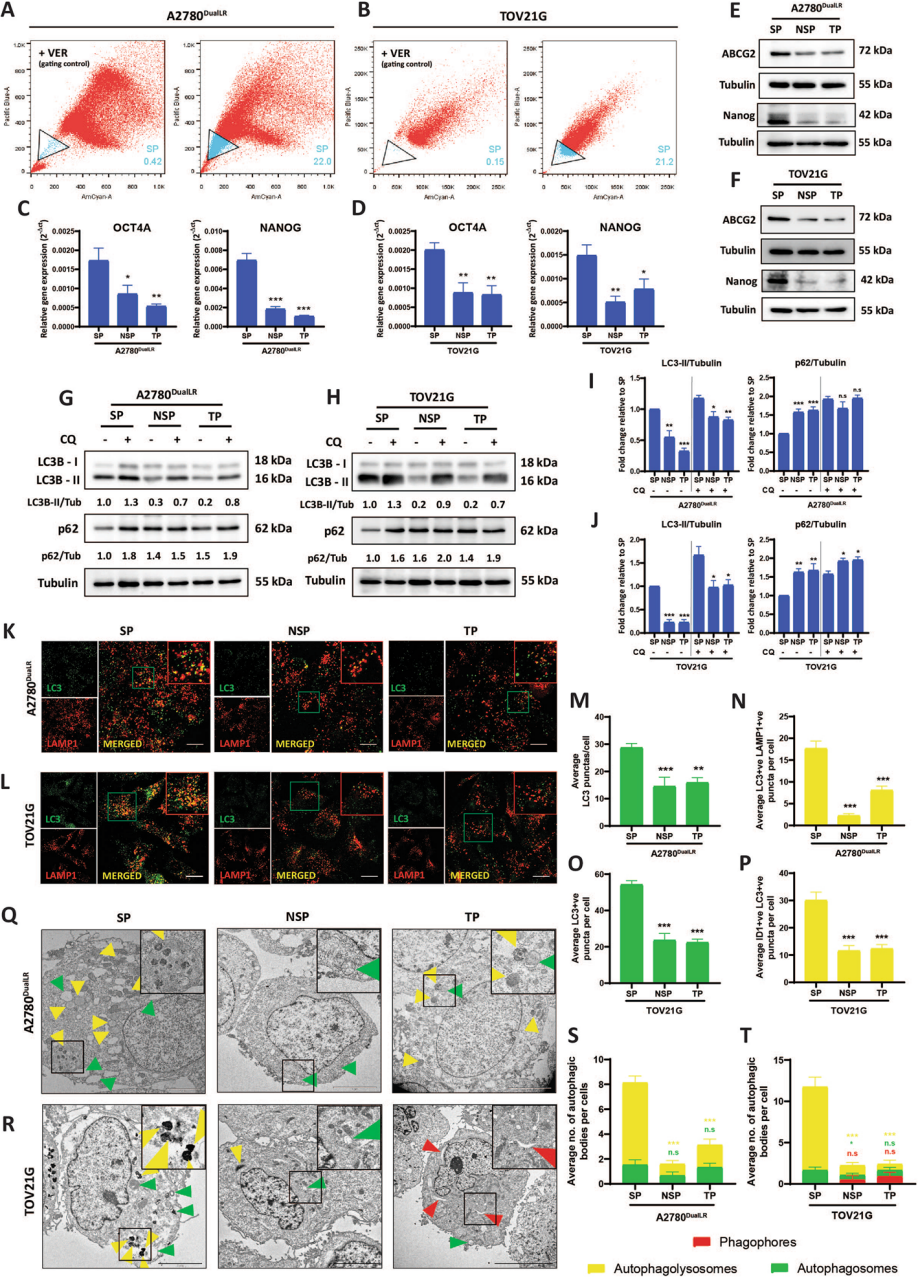
Basal autophagy is elevated in ovarian cancer stem cells (SP) compared to non-stem cells (NSP) and total population (TP). **A, B** FACS plots showing percentage of SP cells (**A**) A2780^DualLR^ and (**B**) TOV21G cells. Verapamil (50 μM) was used as a negative control. **C, D** RT-qPCR analysis show increased OCT4A and NANOG transcripts in SP compared to NSP, and TP cell populations of (**C**) A2780^DualLR^ and (**D**) TOV21G cells (n = 3). **E, F** Immunoblot showed increased expression of ABCG2 and NANOG in SP compared to NSP, and TP cell populations of (**E**) A2780^DualLR^ and (**F**) TOV21G cells (n = 3). **G, H** Immunoblot showed increased LC3B-I to LC3B-II conversion and p62 degradation in SP compared to NSP, and TP cells of (**G**) A2780^DualLR^ and (**H**) TOV21G at the basal level. **I, J** Quantification from 3 independent experiments confirmed significantly elevated basal autophagy in SP versus NSP and TP of (**I:** Graphical representation of the normalized ratio of LC3B-II/Tubulin**,** graphical representation of normalized ratio of p62/tubulin) A2780^DualLR^ and (**J:** Graphical representation of normalized ratio of LC3B-II/Tubulin, graphical representation of normalized ratio of p62/tubulin) TOV21G cells. **K-P** Dual immunofluorescence staining of LC3B (green) and LAMP1 (red) and its quantification (≥ 50 cells/group, n = 2) revealed increased LC3B puncta and LC3B + ve/LAMP1 + ve (yellow) colocalized puncta in SP compared to NSP and TP of (**K:** Representative confocal images**, M, N:** Graphical representation of quantification of average LC3B + ve punctas and average LC3B + ve/LAMP1 + ve punctas per cell respectively) A2780^DualLR^ and (**L:** Representative confocal images**, O, P:** Graphical representation of quantification of average LC3B + ve punctas and average LC3B + ve/LAMP1 + ve punctas per cell respectively) TOV21G cells, indicating higher autophagosome formation and fusion respectively. **Q-T** Transmission Electron Microscopy (TEM) and quantification (≥ 12 images/group, n = 2) of the individual and total autophagic structures showed increased late-stage autophagy bodies called autolysosomes (yellow arrow) in SP compared to NSP and TP of (**Q:** Representative images**, S:** Graphical representation of quantification of the total and individual number of autophagic structures) A2780^DualLR^ and (**R:** Representative images**, T:** Graphical representation of quantification of total and individual number of autophagic structures) TOV21G without significant alterations in phagophores (red arrow) or autophagosomes (green arrow), confirming elevated basal autophagy in ovarian CSCs. Data represented as mean ± SEM. Statistical analysis was performed using one-way ANOVA, followed by Dunnett's post-hoc test when comparing multiple groups to a control, * p < 0.05, ** p < 0.01, *** p < 0.001, n.s.- non-significant. Scale bar: 10 μm for A2780^DualLR^, 20 μm for TOV21G cells (confocal images), 5 µm (TEM images). SP: Side Population, NSP: Non-Side Population, TP: Total Population, UT: Untreated, CQ: Chloroquine, SS: Serum Starvation

CSCs were isolated as side population (SP) from cisplatin-resistant ovarian cancer cell lines A2780^DualLR^ (acquired resistance) and TOV21G (intrinsic resistance) using DCV dye exclusion assay [24] (Fig. 1 A, B). Our previous studies [23, 25] showed that the SP fractions of EOC cell lines had upregulated pluripotency genes, increased spheroid forming ability, increased resistance to platinum and taxol, and were tumorigenic at fewer cell implantation in an immune-deficient mouse compared to non-side population (NSP) fraction. We also observed 2–fourfold higher OCT4A and Nanog transcripts in SP compared to NSP and TP (Fig. 1C, D). The Sox2 levels were undetectable in all these populations for both A2780^DualLR^ and TOV21G cells (data not shown). SP fractions of these two platinum-resistant cell lines showed upregulated ABCG2 (a multidrug and efflux transporter) as well as NANOG (a stemness marker) compared to their NSP and TP counterparts indicating their dye efflux ability and CSC nature (Fig. 1 E, F). This together with our previous results indicated that the SP cells were indeed enriched with the CSC population.

To comprehensively evaluate the basal autophagy level, we performed multi-pronged analyses at both cell population and single-cell resolution. At the basal level, immunoblotting revealed increased LC3B-I to LC3B-II conversion (LC3B-II/Tub ratio: 1.0 in SP vs 0.5 ± 0.17 and 0.3 ± 0.20 in NSP and TP) in A2780^DualLR^ cells and (LC3B-II/Tub ratio: 1.0 in SP vs 0.2 ± 0.12 and 0.2 ± 0.12 in NSP and TP) in TOV21G cells. Decreased p62 levels (p62/Tub: 1.0 in SP vs 1.4 ± 0.17 and 1.6 ± 0.18 in NSP and TP) in A2780^DualLR^ cells and (p62/Tub: 1.0 in SP vs 1.6 ± 0.18 and 1.7 ± 0.3 in NSP and TP) in TOV21G cells were observed. Chloroquine treatment resulted in highest accumulation of LC3B-II in the SP cells (LC3B-II/Tub ratio: 1.1 ± 0.09 in SP vs 0.8 ± 0.17 and 0.8 ± 0.09 in NSP and TP of A2780^DualLR^; 1.6 ± 0.32 in SP vs 0.9 ± 0.29 and 1.0 ± 0.23 in NSP and TP of TOV21G). Although the rates of LC3B-II and p62 accumulation (in absence and in presence of CQ) in SP, NSP and TP cells of A2780^DualLR^ and TOV21G were comparable, the highest LC3B-II and lowest p62 levels were seen in SP cells and their further accumulation after blockade suggested that autophagy was higher in these cells compared to NSP and TP cells of A2780^DualLR^ and TOV21G (Fig. 1G-J).

Dual immunofluorescence staining enabled single-cell assessment of the autophagy pathway. Quantification of LC3B + puncta (green), representing autophagosomes, showed significantly more autophagosomes in SP compared NSP and TP cells at baseline in both cell lines (2.0-fold and 1.8-fold higher than NSP and TP respectively in A2780^DualLR^; 2.2-fold and 2.4-fold higher than NSP and TP, respectively in TOV21G). Analysis of LC3B + /LAMP1 + colocalized puncta (yellow), indicating autophagosome-lysosome fusion, revealed the highest degree of fusion in SP cells for both cell lines (7.7-fold and 2.2-fold higher than NSP and TP, respectively of A2780^DualLR^; 2.2-fold and 2.4-fold higher than NSP and TP, respectively of TOV21G). This verifies that increased autophagosome formation coupled with efficient completion of degradation (Fig. 1 K-P).

Ultrastructural characterization by transmission electron microscopy (TEM) revealed a markedly higher number of electron-dense auto-phagolysosomes, representing the late stages of autophagy, in SP cells compared to NSP and TP counterparts for both cell lines (fivefold and 2.6-fold higher than NSP and TP in A2780^DualLR^; 5.2-fold and 4.8-fold higher than NSP and TP, respectively in TOV21G). No significant differences were observed in number of autophagic intermediates like phagophores or autophagosomes between all the populations (Fig. 1 Q-T).

These multiple approaches establish ovarian CSCs (SP) maintain higher basal autophagy than non-CSCs (NSP), supporting a functional role in preserving the stem-like state.

### Autophagy induction elicits ID1 degradation and CSC differentiation

**Fig. 2:**
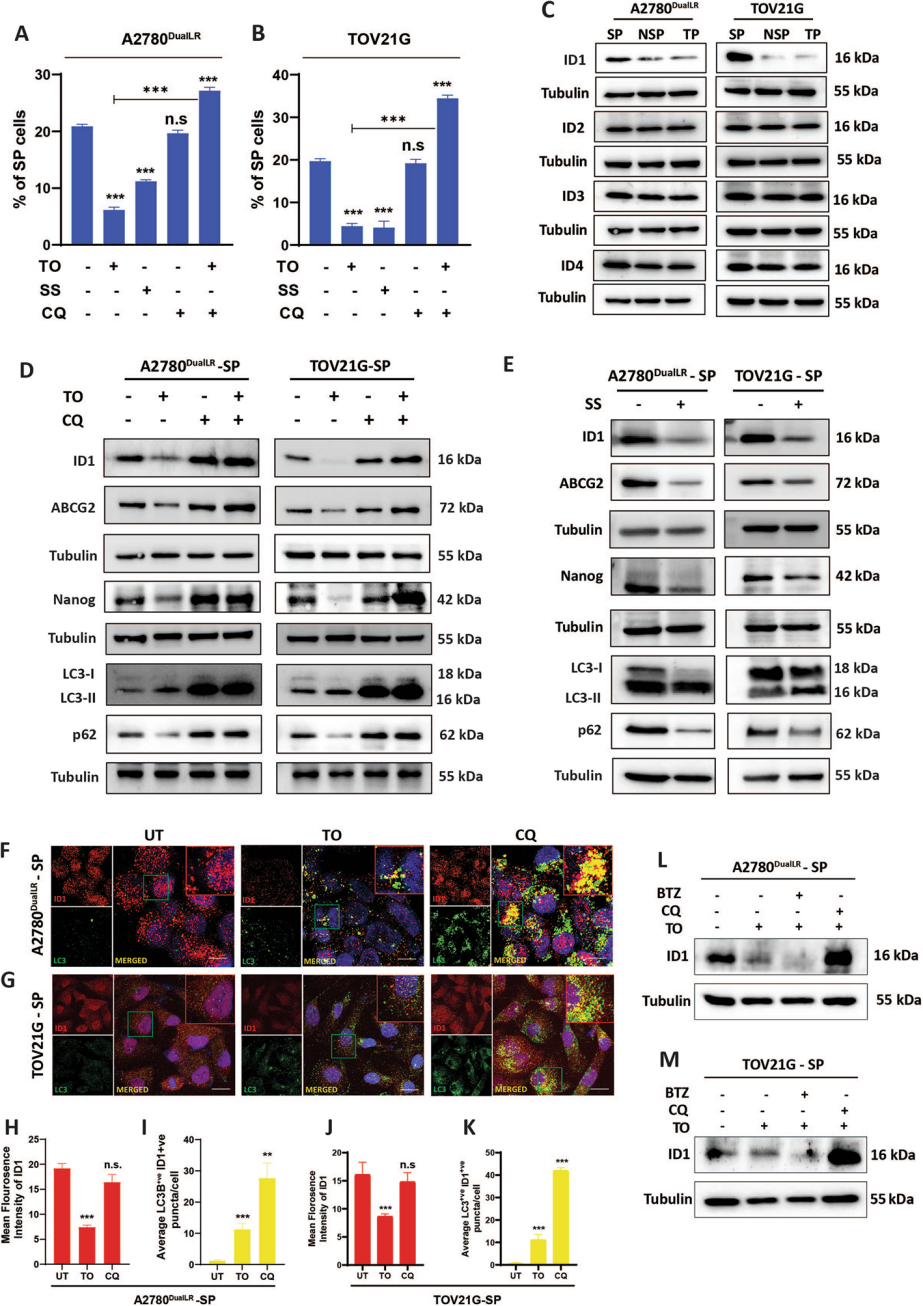
ID1 protein is selectively degraded via autophagy in ovarian CSCs. **A, B** Graphical analysis of Flow cytometry showing a decreased percentage of SP cells with autophagy induction by Torin1 and serum starvation but not with autophagy blockade by CQ. Simultaneous induction and blockade of autophagy (TO + CQ; SS + CQ) partially rescued the effects mediated by autophagy induction (TO and serum starvation) alone showing increased percentage of SP cells and indicating that the observed effects are autophagy induction-specific (**A:** Graphical representation of changes in the percentage of SP cells after autophagy modulation) A2780^DualLR^ cells and (**B:** Graphical representation of changes in the percentage of SP cells after autophagy modulation) TOV21G cells (n = 3). **C** Immunoblotting revealed higher levels of ID1, specifically in SP, compared to NSP and TP of A2780^DualLR^ and TOV21G cells (n = 3). **D** Immunoblot showing that autophagy induction by Torin 1 treatment decreased ID1, ABCG2, and NANOG levels while autophagy blockade by CQ alone showed no significant increase in ID1. Simultaneous induction and blockade of autophagy (To + CQ) showing accumulation of ID1, Nanog and ABCG2 in A2780^DualLR^-SP and TOV21G-SP cells which indicates the rescue effects, mediated by autophagy induction (TO) alone and thereby confirming that the observed effects are autophagy induction-specific. Accumulation of LC3B-II and p62 by CQ which is otherwise reversed after TO treatment increased further after TO + CQ treatment confirming simultaneous induction and blockade in SP populations of both the cell lines (n = 3). **E** Immunoblots showing that autophagy induction by serum starvation also leads to a decrease in ID1 levels with concomitant decrease in ABCG2 and NANOG levels in A2780^DualLR^-SP cells and TOV21G-SP cells. Increased LC3I to LC3II conversion and reduced p62 levels after serum starvation indicated higher autophagy for SP populations of both the cell lines (n = 3). **F-K** Dual immunofluorescence staining of LC3 (green) and ID1 (red) and its quantification (≥ 50 cells/group, n = 2) indicated autophagy induction decreased total ID1 levels and increased colocalization (yellow) with LC3B + puncta in the SP cells of (**F:** Representative images**, H:** Graphical representation of Mean Fluorescence Intensity of ID1**, I:** Graphical representation of average number of LC3 + ve/LAMP1 colocalized punctas per cell) A2780^DualLR^ and (**G:** Representative images**, J:** Graphical representation of Mean Fluorescence Intensity of ID1**, K:** Graphical representation of average number of LC3 + ve/LAMP1 colocalized punctas per cell) TOV21G cells. **L, M** Immunoblot confirming autophagy (CQ), but not proteasome (BTZ: Bortezomib) blockade rescued the Torin 1 treated levels of ID1 in SP cells of (**M**) A2780^DualLR^ and (**N**) TOV21G (n = 2). Data represented as mean ± SEM. Statistical analysis was performed using one-way ANOVA, followed by Dunnett's post-hoc test when comparing multiple groups to a control, or Bonferroni post-hoc test when comparing all groups to each other, whichever is appropriate, * p < 0.05, ** p < 0.01, *** p < 0.001, n.s.- non-significant. SP: Side Population, NSP: Non-Side Population, TP: Total Population, UT: Untreated, VER: Verapamil, TO: Torin 1, CQ: Chloroquine, BTZ: Bortezomib, SS: Serum Starvation

Tumor cells in in vitro culture or in in vivo tumor environment constantly experience stresses that are known to alter their autophagy rates. To determine how autophagy modulation impacts the stem-like state, we adapted two different modes of autophagy induction, one by blocking mTOR pathway and another through serum starvation. Interestingly, Torin 1 treatment selectively reduced the SP fraction in both cell lines (3.4-fold decrease in A2780^DualLR^ cells and 4.4-fold decrease in TOV21G cells) indicating that enhanced autophagy can trigger CSC differentiation. Similarly, serum starvation also led to decrease in the percentage of SP cells (1.8-fold in A2780^DualLR^ cells and 4.8-fold in TOV21G cells). In contrast, chloroquine (CQ) that inhibit late-stage autophagy did not significantly alter the SP population (from 20.8 ± 1.11% to 19.6 ± 1.60% in A2780^DualLR^ cells and from 19.7 ± 1.42% to 19.2 ± 2.24% in TOV21G cells). When cells were treated simultaneously with both Torin1 and CQ, the effects mediated by TO reversed and a significant increase was seen the percentage of SP cells (1.3-fold in A2780^DuallLR^ and 1.7-fold in TOV21G cells compared to untreated cells) indicating that the decrease in percentage of SP cells after TO treatment is autophagy induction specific (Fig. 2 A-B, Fig S2 A, D). Thus, both pharmacological and non-pharmacological induction of autophagy reduced the stem cell like SP population for both A2780^DualLR^ and TOV21G cells.

Among potential autophagy substrates directing cell fate transitions, the inhibitor of differentiation (ID) family of transcriptional regulators are unique since degradation of IDs promote differentiation of stem cells [28]. These ID proteins are generally degraded via ubiquitin-proteosome pathway and there is only one report exist in literature suggesting autophagy mediated degradation of IDs in neuroblastoma [29]. We hypothesized that degradation of IDs by induced autophagy might free up their binding partner/s which are then able to transcriptionally modulate CSC differentiation as well as drug uptake (influx and/or efflux). Such events comprehensively impart a transition from a pluripotent, stemness, drug resistant state to a more differentiated drug sensitive cellular state.

RT-qPCR profiling for ID genes (ID1-ID4) was performed and transcripts showing highest fold change were considered. The analysis revealed that ID1 had the highest upregulation (in terms of fold change relative to TP cells) in SP cells (~ 3.6-fold and ~ twofold in A2780^DualLR^-SP and TOV21G-SP respectively) compared to NSP and TP cells (Fig. S1 A, B).

Subsequently, when expression of all the four mammalian IDs were analyzed, ID1 expression was observed to be selectively higher in SP cells compared to NSPs in both cell lines whereas ID2-4 levels were more or less equivalent (Fig. 2 C).

Autophagy induction by Torin 1 and serum starvation in SP cells triggered massive ID1 degradation in parallel with the decline in ABCG2 and NANOG whereas autophagy blockade by chloroquine resulted in modest accumulation of ID1 along with ABCG2 and NANOG (Fig. 2 D and E). Intriguingly, further accumulation in ID1, Nanog and ABCG2 was observed by simultaneous treatment with CQ and Torin 1 in SP cells of both cell lines with more prominent accumulation in the TOV21G cells (Fig. 2 D).

Dual immunostaining of ID1 (red) and LC3B (green) revealed that at the basal level, ID1 was localized in the nucleus as well as the cytoplasm. Induction of autophagy (characterized by increased LC3B puncta compared to untreated conditions), led to an overall decrease (2.5-fold in A2780^DualLR^ -SP and 2.3-fold in TOV21G-SP) in the levels of ID1 (Fig. 2 F, G, H and J), and redistributed from the nucleus to the cytoplasm, where it was observed to be predominantly colocalized (ninefold in A2780^DualLR^ -SP and 18-fold in TOV21G-SP compared to the untreated conditions) with increased LC3B + ve puncta (Fig. 2 F, G, I, and K). Blocking autophagy led to an overall non-significant decrease in ID1 levels in A2780^DualLR^ -SP cells and TOV21G-SP cells compared to untreated conditions. As expected, the cytoplasmic ID1 showed the highest colocalization (22-fold in A2780^DualLR^ -SP and 75-fold in TOV21G-SP) with LC3B + ve punctas after autophagy blockade (characterized by highest accumulation of LC3B + ve puncta due to blockade) compared to untreated conditions (Fig. 2 F-K).

Rescue experiments in SP cells confirmed chloroquine, but not the proteasome inhibitor bortezomib, could restore ID1 levels upon autophagy induction (Fig. 2 L, M). This verifies selective autophagic turnover of ID1 in SP cells.

Together this data positions ID1 as an autophagy substrate directing ovarian CSC fate that is preferentially degraded when autophagy exceeds a certain threshold required for maintenance.

### ID1 depletion elicits loss of stemness properties and enhances chemosensitivity

To directly examine phenotypic effects of ID1 loss, it was depleted using pharmacological (AGX51 inhibitor; Fig. S1 C-E) and genetic [lentiviral shRNA: one depleting all ID proteins (sh PAN-ID) and the other exclusively targeting ID1(sh ID1)] approaches (Fig. S1 F, G). This dual knockdown approach enabled us to define the distinct versus overlapping roles of ID1 within the ID family.

**Fig. 3:**
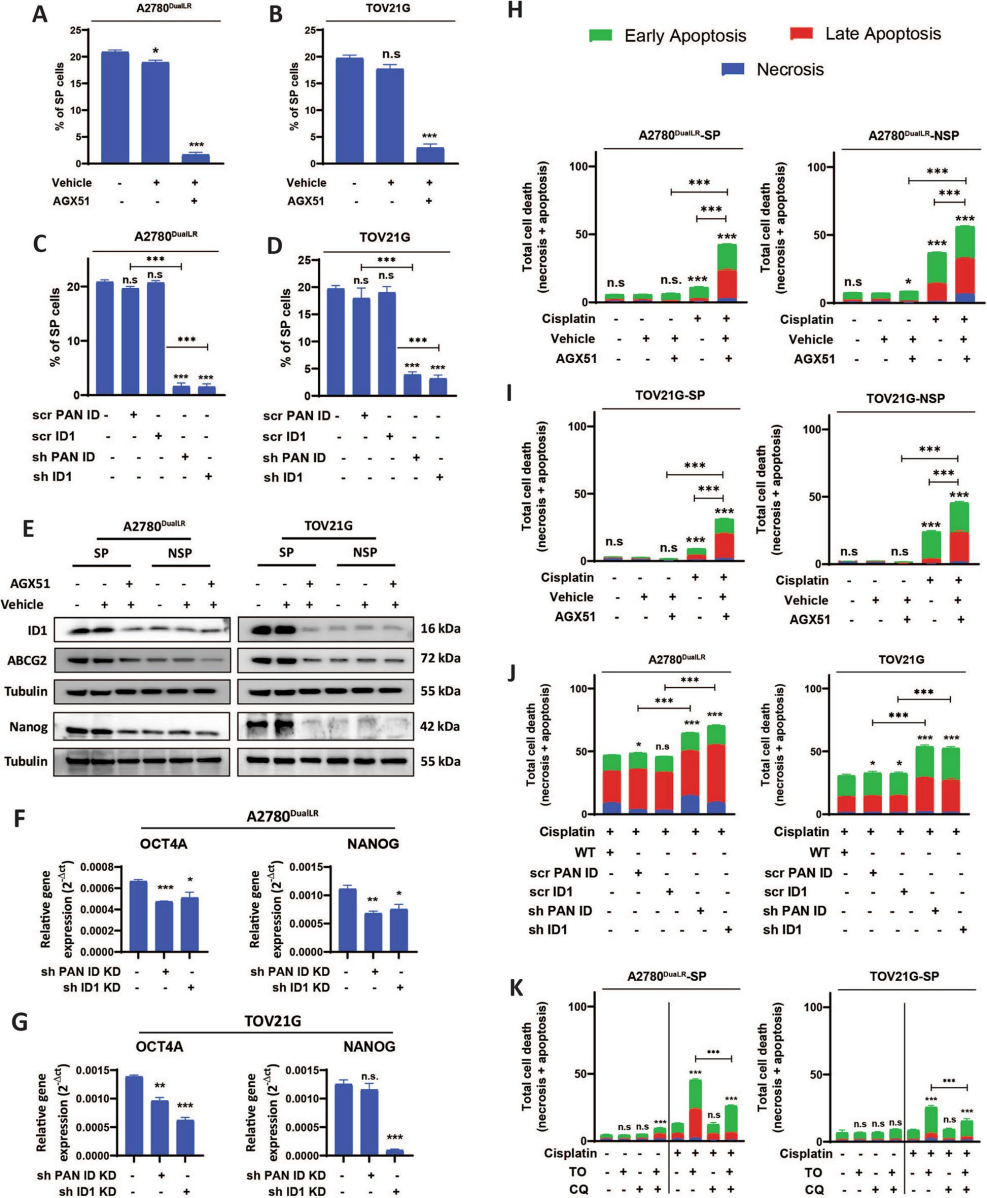
ID1 downregulation decreases stemness and enhances chemosensitivity. **A, D** Graphical analysis of Flow cytometry analysis showing a decreased percentage of SP cells after pharmacological (AGX51, 40 μM) and genetic (shRNA: shPAN-ID and shID1) knockdown of ID proteins in (**A, B**) A2780^DualLR^ and (**C, D**) TOV21G cells. Vehicle controls for AGX51 (0.4% DMSO) showed negligible decrease compared to untreated cells, whereas scrambled controls (scr PAN ID and scr ID1) showed no significant change compared to non-transduced cells (n = 3). **E** Immunoblot analysis of ID1, ABCG2, and NANOG expression in SP cells of A2780^DualLR^ and TOV21G after pharmacological depletion of ID1. Treatment resulted in reduced levels of ID1, ABCG2, and NANOG. Vehicle control showed no apparent changes in the levels of these proteins measured (n = 3). **F** RT-qPCR analysis indicated that genetic ID1 depletion led to downregulation of OCT4A and NANOG transcripts in shPAN-ID and shID1 compared to wild-type cells of A2780^DualLR^ and TOV21G cells (n = 3). **H–L** Annexin V/PI apoptosis assay showed that depletion of ID1 enhanced the cytotoxic effects of cisplatin, causing higher cell death in SP, NSP, and TP cell populations (**H, I:** Pharmacological, **J:** genetic, **K:** autophagy-mediated) of A2780^DualLR^ and TOV21G cells. (**J, K**) Simultaneous induction and blockade of autophagy (TO + CQ) rescued the effects mediated by autophagy induction (TO) alone showing decreased percentages of cell death after cisplatin challenge, contrasting with the effects of Torin1 in A2780^DualLR^-SP cells and TOV21G-SP cells after cisplatin challenge indicating that the observed effects are autophagy induction-specific (n = 3). Minimum of 50,000 events were analyzed per sample. Statistical analysis was performed using one-way ANOVA, followed by Dunnett's post-hoc test when comparing multiple groups to a control, or Bonferroni post-hoc test when comparing all groups to each other, whichever is appropriate. Data represented as mean ± SEM, * p < 0.05, ** p < 0.01, *** p < 0.001, n.s.- non-significant. SP: Side Population, NSP: Non-Side Population, TP: Total Population, UT: Untreated, VER: Verapamil, TO: Torin 1, CQ: Chloroquine

Both AGX51 treatment and shRNA-mediated knockdown consistently decreased the SP fraction of A2780^DualLR^ (11.2-fold after AGX treatment compared to vehicle control and 12 -fold for sh PAN-ID and 13.2-fold for sh ID1 compared to their respective scramble controls scr PAN-ID and scr ID1 respectively) and TOV21G (sixfold after AGX treatment and 12-fold for sh PAN-ID and 14-fold for sh ID1 compared to their respective scramble controls scr PAN-ID and scr ID1 respectively) cells (Fig. 3 A-D; Fig. S4 B, C, E and F). ID1-specific depletion elicited similar responses as PAN-ID knockdown, demonstrating that it is the predominant ID member which regulates ovarian CSC phenotype. Significant reduction in ID1, ABCG2 and NANOG expression was observed in SP cells after pharmacological depletion of ID1 compared to untreated as well as vehicle treated groups. There was no significant difference observed between untreated and vehicle control (Fig. 3 E). Stemness markers OCT4A and NANOG were also decreased following genetic ID1 depletion in both the cell lines, except for a non-significant decrease in Nanog expression in sh PAN-ID of TOV21G cells (Fig. 3 F and G).

Functional effects on cisplatin sensitivity were assessed using annexin V/PI staining (Fig. 3 H–K). As anticipated, SP cells demonstrated increased resistance compared to NSP cells, with 3.3-fold and 2.6-fold lower cell death observed in A2780^DualLR^ and TOV21G SP cells, respectively. AGX51 alone exhibited minimal cytotoxicity, inducing 2–8% cell death across both SP and NSP populations. However, the combination of AGX51 and cisplatin elicited a synergistic cell death response. In SP cells, this combination significantly enhanced cell death compared to cisplatin alone (42.6 ± 1.26% vs. 11% ± 0.53 in A2780^DualLR^-SP; 35.2 ± 1.076% vs. 12.19 ± 1.16% in TOV21G-SP). The chemosensitizing effect was more pronounced in NSP cells, with 56.3 ± 0.43% and 45.7 ± 0.83% cell death occurring in A2780^DualLR^ and TOV21G respectively after combination treatment, substantially exceeding the 2–37% range observed with single agents (Fig. 3 H, I; Fig S5 A; Fig S6 A).

Genetic knockdown of ID proteins further enhanced cisplatin-induced cell death. In A2780^DualLR^ cells, shPAN-ID and shID1 resulted in 64 ± 0.65% and 70 ± 0.63% cell death respectively, while in TOV21G cells, they led to 54 ± 1.37% and 52 ± 0.77% cell death. These results were significantly higher than their respective scrambled controls (48.6 ± 0.20% and 46 ± 0.62% in A2780^DualLR^; 33 ± 0.94% and 32 ± 0.93% in TOV21G for scr PAN-ID and scr ID1, respectively). Scrambled controls showed minimal differences in cell death compared to non-transduced wild type (47 ± 0.75% in A2780^DualLR^ and 30 ± 1.05% in TOV21G) cells. Notably, ID1 depletion alone induced comparable levels of cell death and chemosensitivity to PAN-ID depletion in both cell lines (Fig. 3 J, Fig S5 B, Fig S6 B).

The combination of cisplatin with Torin1, induced substantial cell death in SP cells (45.4 ± 1.34% in A2780^DualLR^-SP; 25.8 ± 2.03% in TOV21G-SP), significantly higher than cisplatin + chloroquine (CQ) treatment (12.5 ± 1.57% in A2780^DualLR^-SP; 9.4 ± 0.6% in TOV21G-SP). This combination phenocopied the effects of AGX51 and shPAN-ID/shID1-mediated ID1 ablation in SP cells. Interestingly, simultaneous addition of CQ with Torin1 and cisplatin partially rescued cells from cell death (from 45.4 ± 1.34% to 26.6 ± 0.72% in A2780^DualLR^-SP and from 25.8 ± 2.03% to 15.6 ± 1.75% in TOV21G-SP cells) (Fig. 3 K, Fig S5 C, Fig S6 C,). This rescue effect suggests that the observed cytotoxicity is largely mediated through autophagy induction, as simultaneous blocking autophagy with CQ after autophagy induction significantly attenuated the cell death induced by the Torin1 + cisplatin combination.

Dose–response cell viability assays further demonstrated a marked decrease in viability and threefold reduction of IC50 values after shRNA-mediated PAN-ID/ID1 depletion in both cell lines (shPAN-ID and shID1cells: 341.2 ng/mL and 321.4 ng/mL respectively of A2780^DualLR^ cells; 372.3 ng/mL and 380.4 ng/mL respectively of TOV21G cells compared to wild type cells: 1005 ng/mL for A2780^DualLR^ and 1162 ng/mL for TOV21G cells) (Fig. S2 A, B, C and D), indicating increased chemosensitivity.

Thus, both CSC and non-CSC compartments are impacted by ID1 loss, with the greatest chemo-sensitizing effect occurring in combination with cisplatin treatment.

### Insilico predictive modeling identifies TCF12 as a top ID1 interacting protein

**Fig. 4:**
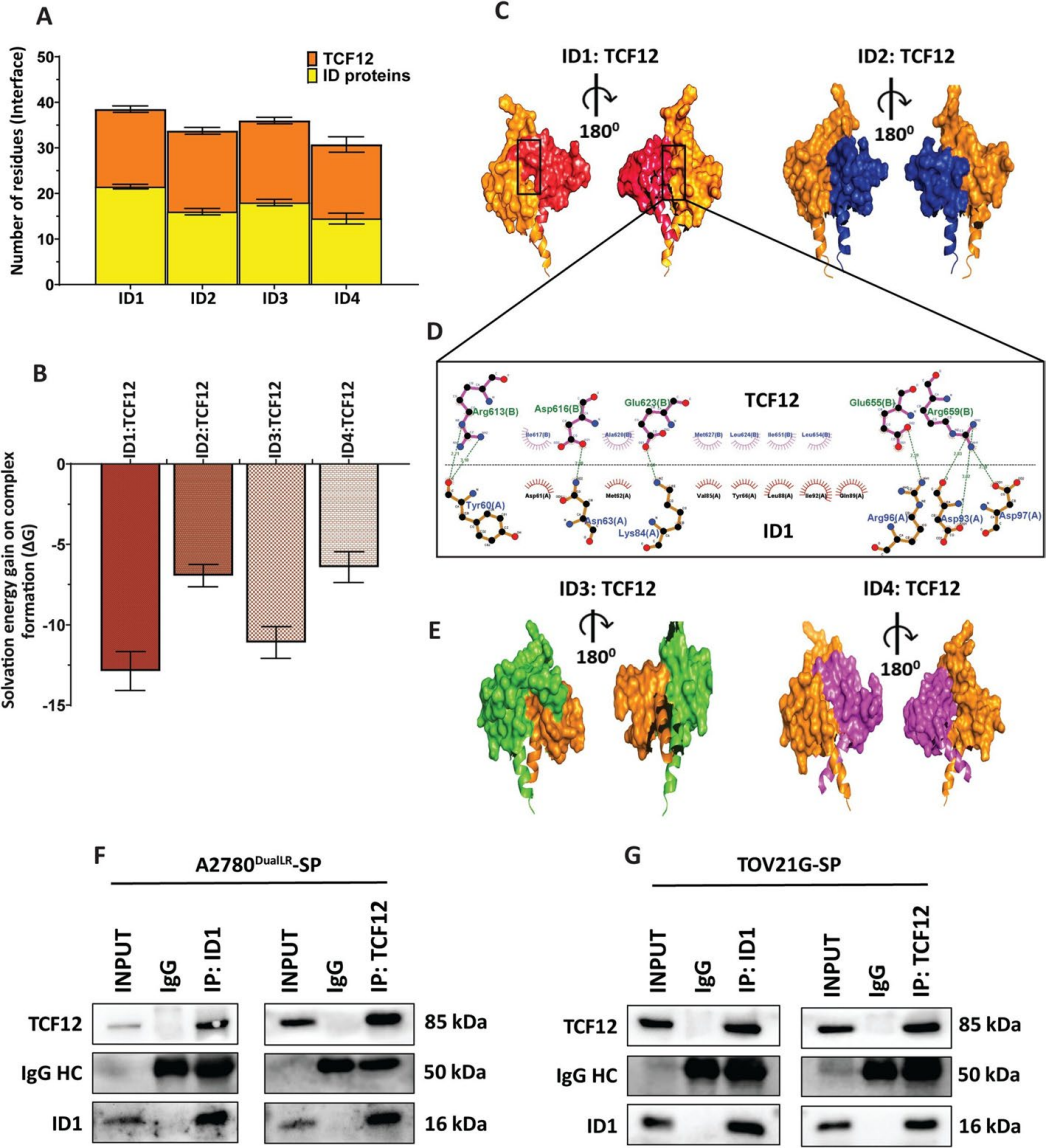
In silico modeling predicted TCF12 as a top interacting protein partner of ID1. **A** Graph showed the total number of residues involved in stabilizing the protein–protein interaction dimers between ID1-ID4 family members and transcription factor TCF12.** B** Graph indicating the thermodynamic feasibility (ΔG values) of dimer interactions between ID1-4 and TCF12. **C** Representative 3D structural models of the dimer complexes formed between ID1-2 family members and TCF12. **D** Ligand–protein interaction plot of the ID1:TCF12 dimer interface highlighting key stabilizing interactions. **E** Representative 3D structural models of the dimer complexes formed between ID3-4 family members and TCF12. **F, G** Co-immunoprecipitation assays demonstrating physical interaction between endogenous ID1 and TCF12 proteins in SP cells isolated from (**F**) A2780^DualLR^ and (**G**) TOV21G cells. Reciprocal immunoprecipitation with anti-ID1 and anti-TCF12 antibodies confirmed the presence of ID1-TCF12 interactions in both cell lines. IgG isotype control was included to confirm specific antibody binding. (n = 2 independent experiments). SP: Side Population

ID proteins lack intrinsic DNA binding domain yet mediate transcriptional repression, through protein–protein interactions [11]. To identify probable ID1 binding partners involved in chemo-resistance (cisplatin uptake or SLC31A1 regulation), we performed in silico predictive modeling. To identify candidate ID1 interactors involved in SLC31A1 regulation, we performed an in silico predictive analysis using STRING (Fig. S3 A) and Agile Protein–Protein Interaction Network (Fig. S3 B) platforms. Preliminary analysis of ID1 interactors revealed a strong association of ID1 with the E family of proteins. E-proteins consisting of TCF3, TCF4, and TCF12 are transcriptional activators that bind DNA at E-box (CANNTG) motifs as homodimers or heterodimers along with other HLH proteins. To elaborate on the functional relevance of ID proteins, in particular ID1, we utilized publicly available 3-dimensional structures of ID proteins. Subsequent docking by HADDOCK 2.2 of ID proteins with the E proteins family was performed, and an in-depth analysis of the interacting interface was done. Among all the ID proteins, ID1 interaction with the E-proteins family showed the highest HADDOCK score of which the ID1:TCF12 interaction was most stable, as ~ 40 residues were involved in stabilizing the dimer (Fig. 4 A). This was corroborated by ~ 130 atoms engaged in stabilizing the ID1:TCF12 dimer (Fig. S3 C).

Further, the thermodynamic stability of ID1:TCF12 interaction was observed by highly negative ΔG values of the dimer (Fig. 4 B). We also found that the ID3:TCF12 interaction was thermodynamically feasible, suggesting the redundant role of ID1 and ID3 proteins. However, ID2 and ID4 interaction with TCF12 was thermodynamically unfavorable. Similar to our previous observations, we found both ID1 and ID3 interaction with other E proteins family of proteins to be the most thermodynamically favorable due to the maximum number of residues and atoms involved in stabilizing the dimers (Fig. S3 G-O). This further highlights the functional redundant roles of ID1 and ID3 proteins.

The conventional zipper-like motif required for proper ID protein function was observed only in ID1 and ID3 interaction with TCF12 in the 3D dimer structures of IDs:TCF12 (Fig. 4 C). Lig-plot analysis of the dimer interface revealed *Tyr-60, Asn-63, Lys-84, Arg-96, Asp-93 and Asp-97* of ID1 forms H-bonding with *Arg-613, Asp-616, Glu-623, Glu-655 and Arg-659* of TCF12 respectively (Fig. 4D). The number of H-bonding between other ID proteins with TCF12 decreased dramatically (Fig. S3 D-F).

To validate this computational prediction, co-immunoprecipitation (co-IP) experiments were performed in ovarian cancer SP cells to identify the endogenous protein–protein interactions. Using an ID1 antibody, we could successfully pull down TCF12, confirming a physical interaction between native ID1 and TCF12 in A2780^DualLR^-SP and TOV21G-SP (Fig. 4 F and G). Similarly, ID1 was also present along with TCF12, when a TCF12 antibody was used for pull down experiment. Together, computational prediction coupled with biochemical co-IP verification identifies TCF12 and ID1 as key interacting proteins.

### Autophagic ID1 degradation releases TCF12 from repression to activate SLC31A1

**Fig. 5:**
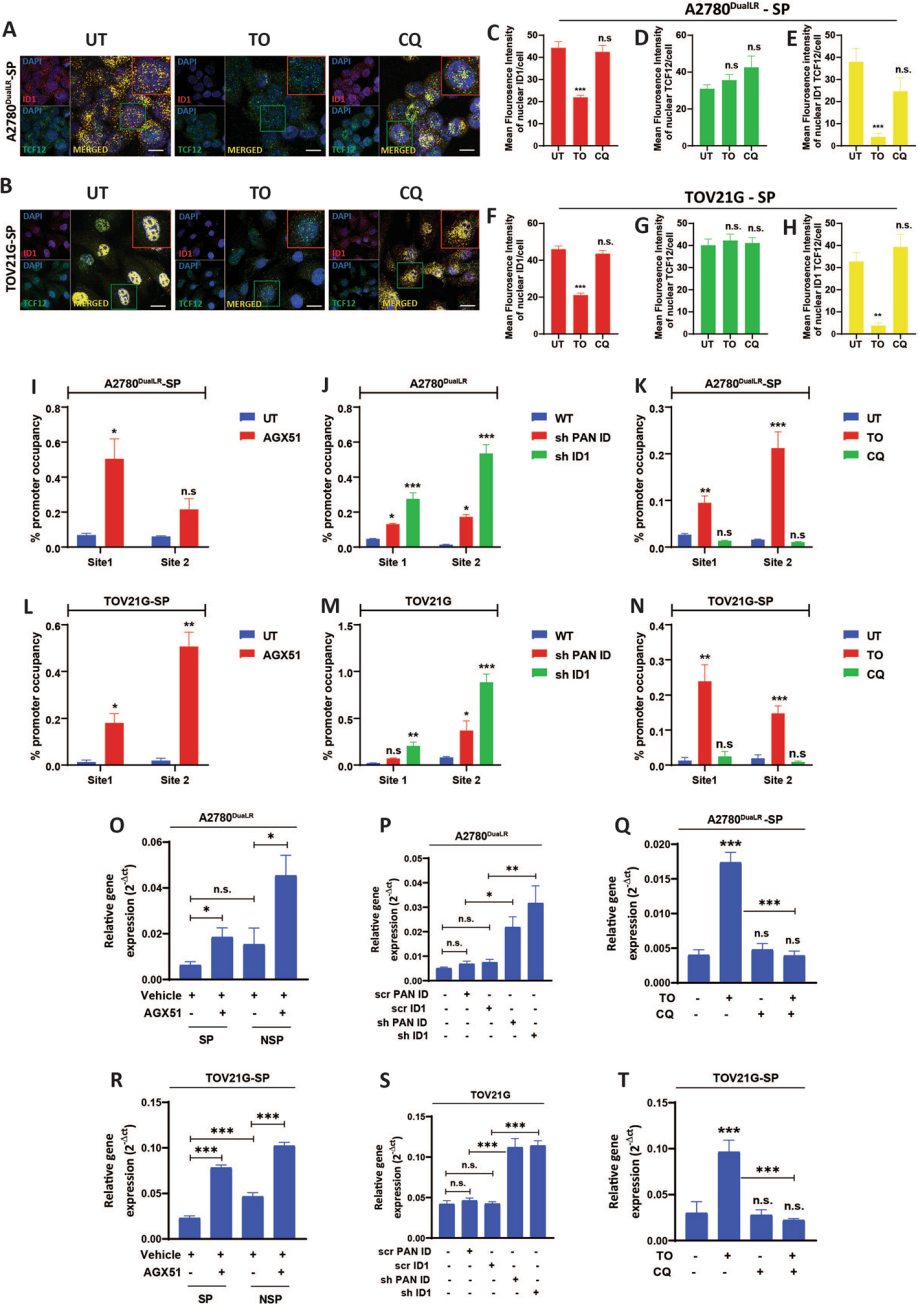
Autophagic degradation of ID1 released TCF12 to activate SLC31A1 promoter. **A-H** Dual immunofluorescence staining of ID1 (red) and TCF12 (green) and their quantification (≥ 50 cells/group, n = 3) indicated that autophagic degradation of ID1 reduces nuclear ID1/TCF12 colocalization (yellow), suggesting that TCF12 was freed from the repression of ID1 in SP cells of (**A:** Representative confocal images**, C:** quantification of nuclear mean fluorescence intensity [mNFI] of ID1**, D:** quantification of mNFI of TCF12**, E:** quantification of mNFI of ID1/TCF12 colocalized puncta) A2780^DualLR^ and (**B:** Representative confocal images**, F:** quantification of mNFI of ID1**, G:** quantification of mNFI of TCF12**, H:** quantification of mNFI of ID1/TCF12 colocalized puncta) TOV21G cells. (**I-N**) ChIP-qPCR data showed increased TCF12 occupancy on SLC31A1 promoter at Site 1 and Site 2 after pharmacological ablation of ID1 in (**I**) A2780^DualLR^-SP and (**L**) TOV21G-SP cells; in shPAN-ID, shID compared to control wild-type (WT) cells of (**J**) A2780^DualLR^ and (**M**) TOV21G cells; after autophagy modulation (TO and CQ) in (**K**) A2780^DualLR^-SP and (**N**) TOV21G-SP cells (n = 2). (**O-T**) RT-qPCR analysis indicated that ID1 depletion led to upregulation of SLC31A1 transcripts in SP, NSP, and TP cell populations of (**O:** Pharmacological, **P:** genetic, **Q:** autophagy-mediated) A2780^DualLR^ and (**R:** pharmacological, **S:** genetic, **T:** autophagy-mediated) TOV21G cells. Simultaneous induction and blockade of autophagy (TO + CQ) rescued the effects mediated by autophagy induction (TO) alone showing decreased levels of SLC31A1, contrasting with the effects of Torin1 in (**P**) A2780^DualLR^-SP cells and (**S**) TOV21G-SP cells thus indicating that the observed effects are autophagy induction-specific (n = 3). (**Q, T**) Scrambled controls of both the cell lines (scr PAN ID and scr ID1) showed no significant changes in the levels of SLC31A1 transcripts compared to non-transduced wild-type cells. Data represents mean ± SEM. Statistical analysis was performed using Student's t-test for comparing untreated and drug-treated group. For comparisons involving multiple groups, one-way ANOVA was used, followed by Dunnett's post-hoc test when comparing multiple groups to a control, or Bonferroni post-hoc test when comparing all groups to each other, whichever is appropriate. * p < 0.05, ** p < 0.01, *** p < 0.001, n.s.- non-significant. Scale bar: 10 μm for A2780^DualLR^, 20 μm–– for TOV21G cells (confocal images). SP: Side Population, NSP: Non-Side Population, TP: Total Population, UT: Untreated, TO: Torin 1, CQ: Chloroquine

Dual ID1/TCF12 immunofluorescence upon autophagy modulation showed autophagy induction reduced nuclear co-localization of these proteins, suggesting release of TCF12 from ID1-mediated repression. TCF12 nuclear levels remained constant after ID1 loss, indicating liberation of TCF12 from ID1 and did not undergo degradation (Fig. 5 A-H).

Cisplatin uptake and retention depend upon the expression levels of copper transporters such as SLC31A1 (influx) or ATP7A, ATP7B (efflux), which determine the platinum sensitivity or platinum resistance of a cell [30]. Interestingly, our in-silico screening predicted two high-scoring TCF12 binding sites on the cisplatin influx transporter SLC31A1 promoter (Fig. S2 E, F). Chromatin immunoprecipitation (ChIP) verified that pharmacological, genetic or autophagic ID1 depletion significantly increased TCF12 occupancy on the SLC31A1 promoter (Fig. 5 I-N). As expected, we saw an increase in the binding of TCF12 (7.4-fold at Site 1 and 3.5-fold at Site 2 in A2780^DualLR^ -SP; 14-fold at Site 1 and 26-fold at Site 2 in TOV21G-SP cells) after AGX-51 treatment. (Fig. 5 I, L) A similar enhancement of TCF12 promoter recruitment was observed following ID1 knockdown. Enhanced promoter occupancy at Site 1 (2.9-fold and 3.3-fold in A2780^DualLR^ and TOV21G cells, respectively) and Site 2 (13.3-fold and 4.5-fold in A2780^DualLR^ and TOV21G cells, respectively) by TCF12 on SLC31A1 promoter was observed in shPAN-ID cells compared to WT cells (Fig. 5 L, O). Similarly, there was an increased– promoter occupancy at Site 1 (sixfold and tenfold in A2780^DualLR^ and TOV21G cells, respectively) and Site 2 (40-fold and 11-fold in A2780^DualLR^ and TOV21G cells, respectively) on SLC31A1 promoter in shID1 cells compared to WT cells (Fig. 5 J, M). In the SP cells, autophagy promotion led to increased binding of TCF12 at Site 1 (3.6-fold in A2780^DualLR^ and 19-fold in TOV21G-SP cells) and Site 2 (13.3-fold in A2780^DualLR^ and 7.7-fold in TOV21G-SP cells). Blocking autophagy led to a twofold decrease in binding of TCF12 at site 1 in A2780^DualLR^, whereas there was non-significant increase at the same site in TOV21G-SP. Similarly, for Site 2, there was a 1.5-fold decrease in TCF12 promoter occupancy in A2780^DualLR^ -SP cells and non-significant decrease in TOV21G-SP cells (Fig. 5 K, N).

In line with this enhanced TCF12 transcriptional activation, RT-qPCR analysis showed ID1 depletion also increased SLC31A1 transcript levels. Treatment with AGX51 led to increased SLC31A1 transcript levels in both SP and NSP cells of A2780^DualLR^ (threefold increase respectively) and TOV21G (3.3-fold and 2.2-fold increase respectively) (Fig. 5 0, R). shPAN-ID and shID1 cells also had increased SLC31A1 transcripts (3.5-fold and 4.4-fold increase compared to scr PAN-ID and scr ID1 in A2780^DualLR^; 2.4-fold and 2.7-fold increase compared to scr PAN-ID and scr ID1 in TOV21G, respectively). The scrambled shRNAs did not exhibit any significant difference compared to non-transduced wild type cells. (Fig. 5 P, S) Similarly, autophagy mediated ID1 degradation increased SLC31A1 transcripts by 4.25-fold in A2780^DualLR^ -SP cells and 3.2-fold in TOV21G-SP cells, while chloroquine treatment did not significantly alter SLC31A1 levels. Surprisingly, SLC31A1 transcript level showed a non-significant decrease with simultaneous induction of autophagy with Torin1 and blockade with CQ for both the cell lines suggesting that the autophagy induction mediated upregulation of SLC31 was reversed as a result of simultaneous autophagy activation and blockade and therefore the observed effects are autophagy specific (Fig. 5 Q, T).

Taken together, this demonstrates autophagic turnover of ID1 frees TCF12 from repression, enabling direct transactivation of SLC31A1 at both the promoter binding and mRNA expression level.

### ID1 suppression enhances SLC31A1 expression and intracellular cisplatin levels

**Fig. 6:**
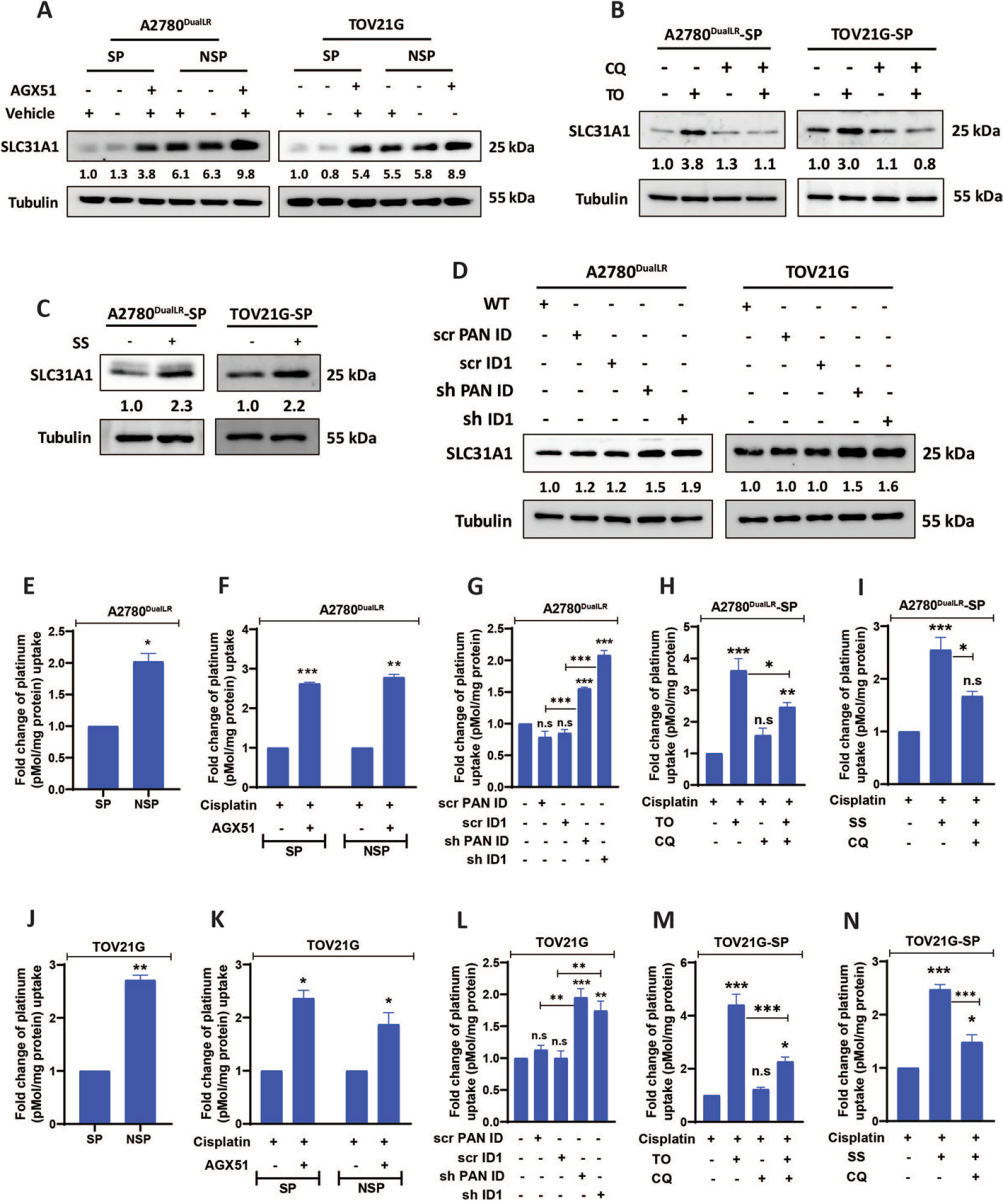
Upregulation of SLC31A1 leads to increased cellular uptake of cisplatin. **A** Immunoblot analysis of SLC31A1 protein levels in A2780^DualLR^ and TOV21G cells. SP cells showed the lowest SLC31A1 expression compared to NSP and TP counterparts. ID1 depletion by AGX51 treatment increases SLC31A1 levels in both SP and NSP cells, with the highest increase observed in NSP cells. Vehicle control (0.4% DMSO) showed no apparent changes in the levels of proteins measured (n = 2). **B** Immunoblot indicated that autophagy-mediated depletion of ID1 by Torin1 treatment led to upregulation of SLC31A1 protein. However, simultaneous treatment with Torin 1 and CQ showed decrease in SLC31A1 protein levels compared to (TO alone) treated group in both A2780^DualLR^ -SP cells and TOV21G-SP cells indicating that the observed effects are autophagy induction-specific (n = 3).** C** Immunoblot showing significant increase levels of SLC31A1 protein levels after serum starvation in both A2780^DualLR^ -SP cells and TOV21G-SP cells (n = 3). **D** Immunoblot indicating that genetic – shPAN-ID and shID1depletion of ID1 also leads to increased levels of SLC31A1 protein with highest increase sh ID1 cells of both A2780^DualLR^ cells and TOV21G cells. Scrambled controls of both the cell lines (scr PAN ID and scr ID1) showed no significant changes in the levels of SLC31A1 protein compared to non-transduced wild-type cells (n = 3). **E-N** ICP-MS analysis to quantify normalized intracellular platinum levels showed lower basal platinum accumulation in SP compared to NSP cells of (**E**) A2780^DualLR^ and (**J**) TOV21G cells. Various strategies of ID1 depletion led to enhanced platinum uptake in both SP and NSP cells of (**F:** pharmacological, **G:** genetic—shPAN-ID and shID1**, H-I:** autophagy-mediated) A2780^DualLR^ and (**K:** pharmacological, **L:** genetic—shPAN-ID and shID1**, M–N:** autophagy-mediated) TOV21G cells. Simultaneous induction and blockade of autophagy rescued the effects mediated by autophagy induction by Torin1 and serum starvation, resulting in a significant decrease in cisplatin accumulation compared to autophagy induction alone (Torin1 treatment and serum starvation) indicating that the observed effects are autophagy induction-specific. These effects are shown in (**H-I**) for A2780^DualLR^-SP cells and (**M–N**) for TOV21G-SP cells. Scrambled controls of both the cell lines (scr PAN ID and scr ID1) showed no significant accumulation of cisplatin compared to non-transduced wild-type cells (n = 3). Data are mean ± SEM. Statistical analysis was performed using Student's t-test with Welch’s Correction for comparing two groups for data with unequal variances. For comparisons involving multiple groups, one-way ANOVA was used, followed by Dunnett's post-hoc test when comparing multiple groups to a control, or Bonferroni post-hoc test when comparing all groups to each other, whichever is appropriate. * p < 0.05, ** p < 0.01, *** p < 0.001, n.s.- non-significant. SP: Side Population, NSP: Non-Side Population, TP: Total Population, UT: Untreated, TO: Torin 1, CQ: Chloroquine, SS: Serum Starvation

The copper transporter SLC31A1 regulates cisplatin influx and sensitivity [31]. Basal SLC31A1 protein levels were markedly lower in the drug-resistant SP versus NSP cells (Fig. 6 A). ID1 depletion via all approaches—autophagic, pharmacological, and genetic—upregulated SLC31A1 transcripts (Fig. 5 O-T) and protein in both populations (Fig. 6 A-D, Quantification of all the blots is presented in Fig S7 A-H). Strikingly, inductively coupled plasma mass spectrometry (ICP-MS) quantified significantly increased intracellular cisplatin accumulation following ID1 depletion (Fig. 6 E-N). As expected, basal Pt levels were markedly lower in SP cells compared to NSP cells (twofold lower in A2780^DualLR^ SP cells versus NSP cells; 2.7-fold lower in TOV21G SP cells versus NSP cells) (Fig. 6 E, J). This is consistent with the proposed association between cisplatin efflux/reduced influx and the drug-resistant phenotype. Significantly higher Pt accumulation in both SP and NSP cells was observed following pharmacological ID1 degradation with AGX51 compared to control cells (2.6-fold increase in Pt in A2780^DualLR^-SP cells, 2.7-fold increase in A2780^DualLR^-NSP cells; 2.3-fold increase in Pt in TOV21G SP cells, 1.8-fold increase in TOV21G NSP cells) (Fig. 6 F, K). Pt levels also showed a notable enhancement in ID knockdown cells compared to their respective scrambled controls (twofold increase in shPAN-ID cells compared to scr PAN ID1, 2.3-fold increase in sh ID1 cells compared to scr ID1 of A2780^DualLR^ cells; 1.7-fold increase in shPAN-ID cells compared to scr PAN ID1; 1.7-fold increase in sh ID1 cells compared to scr ID1 of TOV21G cells). The scrambled controls showed no significant difference in platinum accumulation compared non-transduced wild-type cells. (Fig. 6 G, L). Augmentation of autophagy by Torin 1 treatment (pharmacological autophagy inducer) significantly increased Pt accumulation in SP cells (3.5-fold increase in A2780^DualLR^ SP cells; 4.3-fold increase in TOV21G SP cells) (Fig. 6 H, M). Similar results were obtained after serum starvation, a non- pharmacological autophagy inducer (2.5-fold increase in A2780^DualLR^ SP cells; 2.4-fold increase in TOV21G SP cells) (Fig. 6 I, N). Interestingly, simultaneous activation of autophagy (by Torin1 or serum starvation) and blockade of autophagy by CQ (TO + CQ; SS + CQ) reduced the autophagy induction (TO or SS) mediated increased platinum uptake by 1.6-fold and 2-fold for A2780^DualLR^-SP and TOV21G-SP respectively in TO + CQ group and 1.5-fold and 1.6-fold for A2780^DualLR^-SP and TOV21G-SP respectively in SS + CQ group. Such a decrease in platinum accumulation after simultaneous induction and blockade of autophagy indicates that the platinum accumulation is autophagy induction specific (Fig 6. H, I, M, N).

Collectively, this data validates a model where ID1 acts as a key switch governing SLC31A1 expression and platinum sensitivity. Autophagic ID1 degradation or direct inhibition lifts repression on SLC31A1, increasing cisplatin uptake and cytotoxicity.

### Patient tumor spheroids link stemness, autophagy, SLC31A1, and platinum sensitivity

**Fig. 7:**
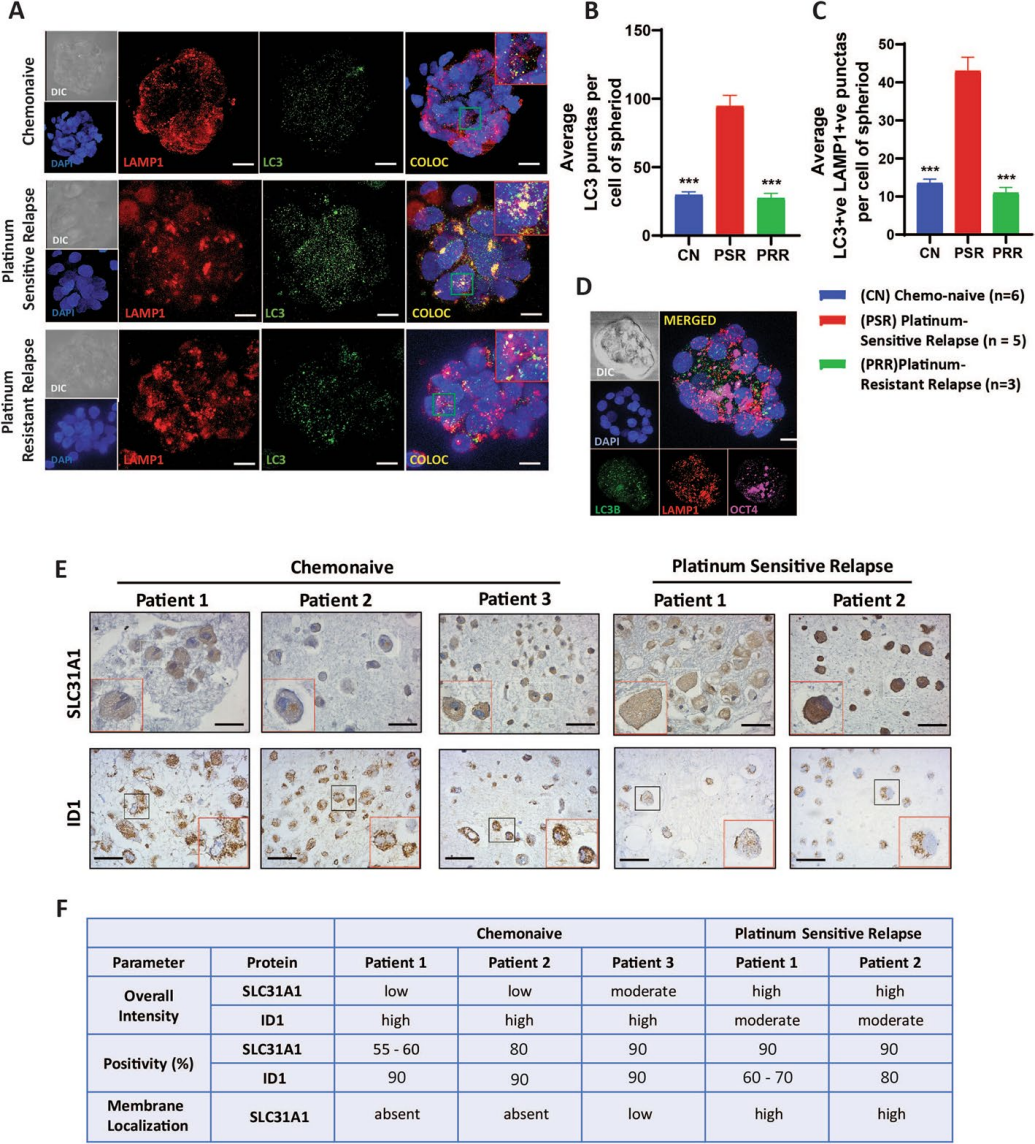
Platinum-sensitive patient spheroids exhibit heightened autophagy, ID1 and SLC31A1 expression. **A** Dual immunofluorescence staining of LC3B (green) and LAMP1 (red) in tumor spheroids derived from the ascitic fluid of chemo-naïve (sample size n = 6 patients; total of 38 spheroids analyzed across all patients), platinum-sensitive relapsed (sample size: n = 5 patients; total of 34 spheroids analyzed across all patients) and platinum-resistant relapsed (sample size: n = 3 patients; a total of 13 spheroids analyzed across all patients) high-grade serous ovarian cancer patients showed increased LC3B + ve punctas that colocalized (yellow) more with LAMP1 in spheroids from platinum-sensitive relapsed cases indicated a higher autophagy. **B** Graphical representation of quantification of average LC3B + ve punctas per cell of a spheroid**. C** Graphical representation of quantification of average LC3B + ve/LAMP1 + ve punctas per cell of a spheroid**. D** Representative image showed positive staining for stemness marker OCT4 in patient-derived spheroids. **E** Representative immunohistochemistry images of SLC31A1 and ID1 staining in cell blocks made from ascites of chemo-naïve and platinum sensitive relapse patients. **F** Tabulated immunohistochemistry (IHC) scores of SLC31A1 and ID1 in cell blocks. Results showed highest intensity and membranous localization of SLC31A1 in platinum-sensitive relapsed patients compared to chemo-naïve patients. Conversely, ID1 expression was lower in platinum-sensitive relapsed patients compared to chemo-naïve patients. Patient-level Means (average of spheroids analyzed per patient) ± SEM obtained from each category were used for statistical analysis. Statistical analysis was performed using one-way ANOVA followed by Dunnett's post-hoc test for multiple comparisons. * p < 0.05, ** p < 0.01, *** p < 0.001, n.s.- non-significant. Scale bar: 5 μm (confocal images), 40 μm (I.H.C images)

High Grade Serous Ovarian Cancer (HGSOC) is characterized for rapid relapse due to acquirement of platinum resistance and is often speculated as CSC driven disease present as tumor spheroids in the malignant ascites [32]. The relapsed HGSOC patients can be characterized into two categories based on the time of relapse and repeat response to cisplatin (platinum resistant relapse and platinum sensitive relapse). To assess the observed association between of autophagy and platinum resistance in clinical cases, we isolated and characterized tumor spheroids for stemness from the malignant ascites of high-grade serous ovarian carcinoma patients, including chemo-naïve, platinum-sensitive or resistant relapses cases. All these spheroids expressed OCT4 at differential levels. Next, dual immunofluorescence staining revealed spheroids from platinum-sensitive relapse cases displayed 3.2-fold higher LC3B punctas (green) compared to chemo-naive and 3.4-fold higher compared to platinum-resistant relapse cases indicating increased autophagy. Further, LC3B + ve/LAMP1 colocalized punctas (yellow) were highest in the platinum-sensitive relapse cases (3.1-fold higher compared to chemo naive and fourfold higher compared to platinum-resistant relapse cases) indicating that the autophagy is indeed getting complete in the spheroids of platinum-sensitive relapse cases (Fig. 7 A-D).

Since, it was not possible to assess SLC31A1 in intact spheroids due to their 3D nature, cell blocks were prepared for some of the cases where cells could be cultured. Unfortunately, we were not able to make cell blocks from spheroids of platinum-resistant relapse cases. Immunohistochemistry analysis of these cell blocks showed the highest SLC31A1 membrane expression and intensity in platinum-sensitive relapsed cases (Fig. 7 E and F). Intriguingly, an opposite trend was observed for ID1 expression which was higher in platinum-sensitive relapse cases compared to chemo naive cases.

Together with the cell line models, this tumor-derived spheroids data although with limited sample size, provides critical validation of role of autophagy controlling CSC fate and drug response, and warrants further biomarker investigation in expanded patient cohorts.

## Discussion

**Fig. 8:**
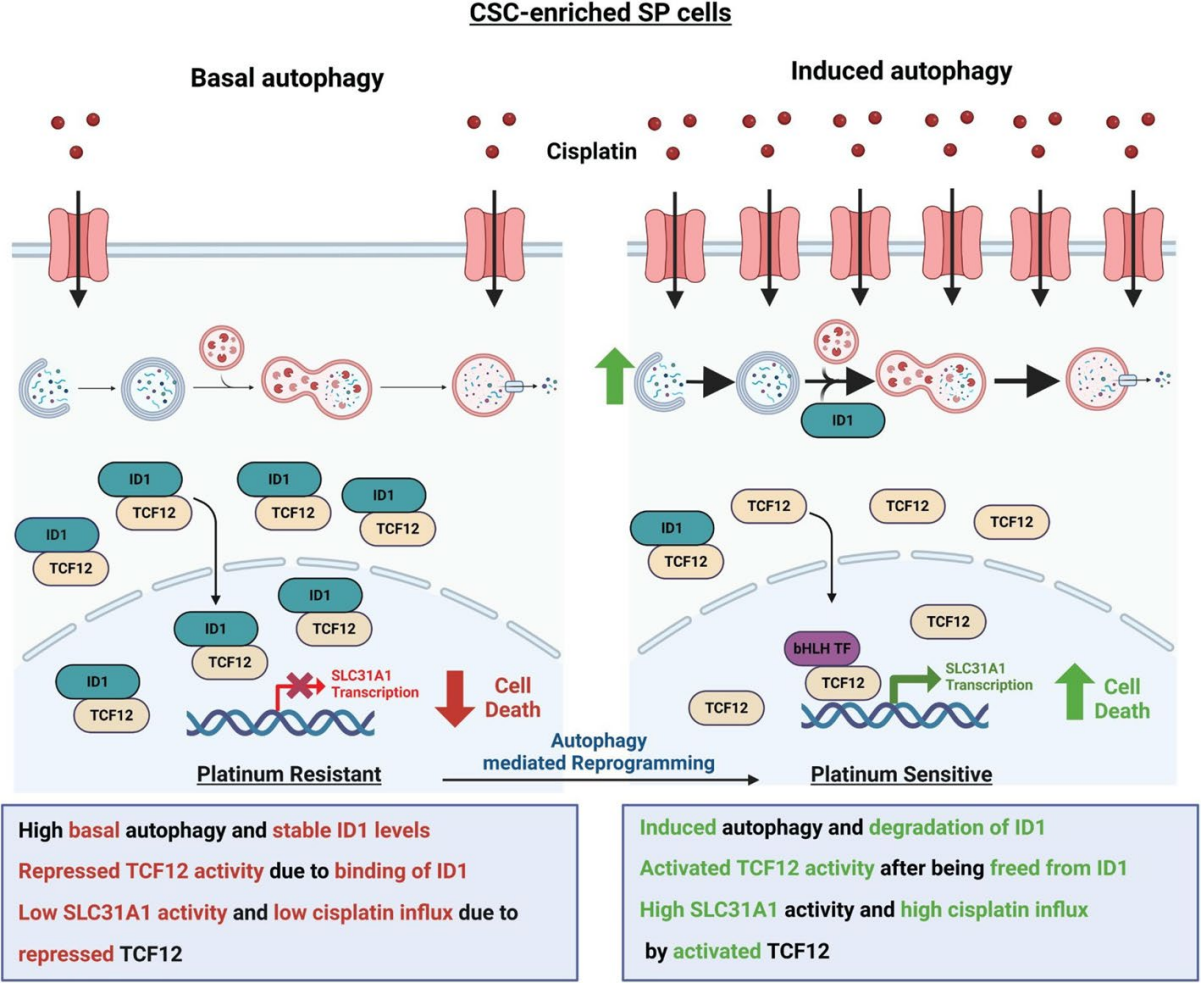
Autophagy-Mediated Regulation of Cisplatin Sensitivity in Ovarian Cancer Stem Cells. **A** Schematic comparison of basal and induced autophagy pathways regulating chemotherapy response in CSC-enriched SP cells. At basal level, high ID1 levels sequester TCF12, reducing SLC31A1 expression and cisplatin influx. Induced autophagy degrades ID1, liberating TCF12 to enhance and upregulate SLC31A1, leading to increased cisplatin sensitivity. This autophagy-mediated reprogramming shifts the cells fate from a platinum-resistant to platinum-sensitive state (Created with BioRender.com)

Autophagy, a catabolic process is primarily utilized by drug resistant cancer cells as a strategic method for survival by connecting multiple cellular properties starting from cancer stem cell (CSC) phenotypes, EMT, tumor growth and recurrence and therapy resistance [4, 5]. The precise molecules and molecular pathways in these connecting nodes are not completely identified and remains a thrust area of current cancer research. Our study elucidates a novel pathway by which targeted autophagic degradation of the transcriptional regulator ID1 enables dynamic control over ovarian CSC fate and cisplatin response. We demonstrate for the first time that promotion of autophagy by both pharmacological and non-pharmacological strategies leads to proteolytic degradation of ID1 in the CSC-enriched side population (SP) of platinum-resistant epithelial ovarian cancer (EOC) cell lines. This liberates the ID1-bound transcription factor TCF12 to transactivate the cisplatin influx transporter SLC31A1, effectively reprogramming the cells from a chemo resistant to chemo-sensitive phenotype (Fig. 8). Analysis of patient tumor spheroids supported links between elevated autophagy, ID1 and SLC31A1 expression, and platinum sensitivity in clinical samples, thereby validating the relevance of these molecular connections in the highly aggressive HGSOC disease.

Our work reveals that ovarian CSCs possess higher basal autophagy compared to the non-CSC population, suggesting a functional requirement of autophagy in preserving the stem-like state. This aligns with several studies in different cancer contexts where autophagy enables cancer cell survival under stressful tumor microenvironments and is important for maintaining self-renewal capacity and tumorigenicity [33–38]. The spatial and temporal autophagy in cancer cells is always dynamic and depends upon the various stresses generated in tumor environment of a growing tumor. Herein, we newly show that further autophagy induction past a critical threshold over the basal level triggers ovarian CSC differentiation, effectively depleting this chemo-resistant pool of cells by promoting conversion into non-CSCs, marked by declining expression of efflux and stemness regulators like ABCG2 and NANOG in SP cells. This contrasts with autophagy blockade where, despite modest accumulation of ABCG2 and NANOG, does not significantly increase the SP fraction overall. However, dual manipulation of autophagy with simultaneous induction as well as blockade leads to accumulation of ID1, and ABCG2 in both cell lines concomitant with the significant increase in percentage of SP cells indicating that the fate of the SP cells is in part controlled by autophagy. These findings underscore the complex role of autophagy in regulating cancer stem-like cells, with induction of autophagy exerting a more pronounced negative effect on SP cell populations than autophagy inhibition.

Autophagy is known to exert precise control over cell fate transitions by degrading select master regulatory stemness proteins like OCT4 [39], SOX2 [40], NANOG [41], MEKK3 [42], Dvl [43], WT1 [44], FOXO3A [45], TWIST1 [46], and Myc, SMAD2/3 [47], β-catenin [48] in a context dependent manner. We identify ID1 as another novel autophagy substrate that can act as a molecular switch regulating ovarian CSC fate, differentiation and chemo-sensitization.

The four members of ID family (ID1-4) share a strong structural homology and act as dominant negative regulators for several basic helix-loop-helix (bHLH) proteins, producing distinct cell and tissue-specific functional effects [10]. Such tissue and stage-dependent differential expressions of ID proteins required for the maintenance of embryonic and tissue stem cells are well documented [49–51]. ID1, in particular, is overexpressed in gastric, ovarian, colon, breast, liver, and other cancer cells, and shown to be associated with cell growth, EMT, cell cycle, metabolic reprogramming and with drug resistance [52]. Till date, role of ID1 in preserving stemness and drug resistance is elucidated in hepatocellular CSCs [53] glioblastoma CSCs [15] and colon CSCs [12]. In ovarian cancer specifically, ID1 expression is associated with poor differentiation, aggressive tumor behavior, and poor clinical outcomes in patients [8, 9]. Meng et al. (2020) had shown that ID1 upregulates autophagy in multiple EOC cell lines through ATF6 activation possibly via a NFkb-IL6-STAT3 pathway and therefore, overexpression of ID1 confers chemo resistance in these EOC cell lines [16]. Their data corroborate with our observation that pharmacological or genetic depletion of ID1 bestowed chemosensitivity and higher ID1 expression along with a high basal autophagy is evident in chemo-resistant CSC population. However, the authors did not attempt to address the influence of autophagy modulation on ID1 stability which is crucial to maintain cellular homeostasis and stemness phenotype in chemo-resistant and stress conditions. They also did not perform any biochemical assay to show the direct influence of ID1 on NF-kB or STAT3 on ATF6. Thus, it is important to dissect out the molecular nodes connecting autophagy, ID1, platinum-resistance using appropriate chemo-resistant cell lines and patients’ samples.

Among our ovarian cancer models, ID1 expression was found to be selectively elevated in the CSC-enriched SP which also exhibit increased autophagy. This may be caused by elevated transcription of ID1 counteracting the basal autophagic degradation. The reason for elevated ID1 expression in our cellular models is currently unknown and currently being investigated. None the less, this positions ID1 as a likely mediator of stem-like traits. Accordingly, ID1 downregulation reduced the SP fraction and stemness markers while enhancing cisplatin-induced cell death.

Our shRNA dual knockdown approach elegantly parsed out the distinct and complementary functions of ID1 and the other ID protein network. Importantly, while both ID1-selective and pan-ID family knockdown reduced ovarian cancer stemness properties, ID1-specific depletion elicited more robust effects on critical endpoints. ID1 knockdown caused a greater reduction in the percentage of SP cells, more potent downregulation of pluripotency markers, and enhanced TCF12 recruitment to the SLC31A1 promoter to a greater extent than pan-ID knockdown. The two approaches showed comparable cisplatin-induced cell death. These results indicate that despite some functional redundancy between ID family members, ID1 is the predominant ID member regulating ovarian CSC identity and plasticity. ID1 depletion alone elicits significant differentiation and chemo-sensitization effects. However, the enhanced intracellular cisplatin accumulation with pan-ID knockdown implies potential involvement of other ID proteins in modulating drug influx through SLC31A1.

Lack of DNA binding domains compel ID proteins to act as dominant negative transcriptional repressors by sequestering class I bHLH E proteins like TCF3, TCF4, and TCF12 [11]. Our in-silico modeling predicted TCF12 as a top ID1 interacting protein, validated by co-immunoprecipitation. We discovered that liberating nuclear TCF12 from ID1 repression enabled transcriptional activation of the cisplatin influx transporter SLC31A1 which led to 2–fivefold increase in intracellular platinum levels directly demonstrating increased drug uptake. Multiple lines of evidence including ChIP assays also verify TCF12 promoter recruitment and SLC31A1 transactivation upon ID1 depletion.

Low SLC31A1 expression is linked with platinum resistance in ovarian and other malignancies [54–58] while higher SLC31A1 expression sensitizes cells to platinum drugs [31, 59–61] We found basal SLC31A1 was reduced in the SP, consistent with its drug-resistant phenotype. Prior reports suggest that SLC31A1 can be modulated by factors like Sp1 [62], HIF2α [63], β-catenin/TCF4 [64], and recently by the histone demethylase JHDM2A [61]. Our work discovered a direct role of ID1-TCF12 axis in cisplatin influx of ovarian CSCs which has never been reported earlier. This interaction may provide a druggable node for altering SLC31A1 expression and platinum sensitivity.

HGSOC, the most prevalent subtype of EOC is infamous for relapse and platinum-resistant characteristics. Among these relapsed patients, a significant number respond (platinum sensitive relapse) to platinum agents again after disease recurrence [32]. The underlying molecular pathway/s of such re-sensitization has not been well understood. Using a small cohort of patient derived tumor spheroids, we showed an inherently higher autophagy as well as higher expression and membranous localization of SLC3A1 in these platinum sensitive relapse patients. We speculate existence of an active ID1-TCF12 axis governed by autophagy is the underlying cause for re-sensitization towards platinum by these patients. While our study provides comprehensive in vitro and ex vivo evidence on these critical aspects of platinum-resistance, there are certain limitations which would need follow up investigation. A through in vivo experiments that would assess pre-clinical evaluation of AGX51 are planned in future. We also plan to extend our observations in a larger cohort of patients and the direct effect of AGX51 in tumor spheroids. Extending this analysis to larger patient cohorts with appropriate molecular assays could further substantiate the potential of ID1 as a biomarker approach to guide platinum re-treatment decisions after relapse.

In this study, we primarily focused on ID1, but acknowledge that other ID family members due to their overlapping functions might have roles in some of the pathways particularly in the NSP population. Further investigations are needed to fully elucidate the roles of ID2-4 which is beyond the scope of this study. Additionally, autophagy regulates cancer cell response to chemotherapy through multiple mechanisms beyond ID1 degradation, including cytoprotection, DNA damage response modulation, oxidative stress regulation, and influence on stemness properties and the tumor microenvironment. These diverse roles highlight the complex interplay between autophagy and drug sensitivity, as demonstrated by studies showing enhanced cisplatin efficacy upon autophagy inhibition in various cancer types [65–67]. Future research should aim to delineate the specific contributions of these autophagy-mediated pathways to chemosensitivity in different cancer contexts. Finally, our study primarily examined short-term responses to autophagy modulation and ID1 inhibition. Long-term effects and potential adaptive responses warrant further investigation.

However, our study also demonstrates that the autophagy-ID1-TCF12-SLC31A1 axis could be developed into a predictive biomarker panel for platinum sensitivity in ovarian cancer. Some of the nodes of this axis such as ID1, autophagy, SLC3A1 transporter are targetable and might aid in development of potential context dependent targetable drugs in future. Thus, the autophagy-ID1-TCF12-SLC31A1 axis represents a promising target for overcoming chemotherapy resistance, and further research in this area could significantly impact patient outcomes.

## Conclusion

Overall, this study defines a novel autophagy-ID1-TCF12-SLC31A1 axis that exerts rapid, targeted control over ovarian CSC fate and drug response. Strategies modulating this regulatory hub could counter chemo-resistance.

### Supplementary Information

Supplementary Material 1.

## Data Availability

All data generated or analyzed during the current study are included in this published article (and its supplementary information files).

## Abbreviations

CQ: Chloroquine
CSC: Cancer stem cell
EOC: Epithelial ovarian cancer
ICP-MS: Inductively Coupled Plasma Mass Spectrometry
ID1: Inhibitor of differentiation 1
LAMP1: Lysosomal-associated membrane protein 1
LC3B (MAP1LC31B): Microtubule-associated protein 1B-light chain 3
Mnfi: Mean Nuclear Fluorescence Intensity
NSP: Non-side population
SLC31A1: Solute Carrier Family 31 Member A1
SP: Side population
SS: Serum Starvation
TCF12: Transcription Factor 12
TP: Total population
