# Supplementary material for: Autophagy-mediated ID1 turnover dictates chemo-resistant fate in ovarian cancer stem cells
Source: J Exp Clin Cancer Res. 2024 Aug 10;43:222. doi: 10.1186/s13046-024-03147-z (PMC11316295; doi:10.1186/s13046-024-03147-z)
Supplement: Supplementary file 1 — Supplementary Material 1. [file 13046_2024_3147_MOESM1_ESM.pdf]

Supplementary Information

Autophagy-mediated ID1 turnover dictates chemo-resistant fate in Ovarian Cancer Stem Cells

Pratham Phadte[1,3], Aniketh Bishnu[1,3], Pranay Dey[2,3], Manikandan M[4], Megha Mehrotra[1,3], Prerna Singh[1,3], Shritama Chakrabarty[1,3,6], Rounak Majumdar[1,3,7], Bharat Rekhi[3,5], Malay Patra[4], Abhijit De[2,3], and Pritha Ray[1,3]*

1 Imaging Cell Signalling & Therapeutics Lab, Advanced Centre for Treatment, Research and Education in Cancer, Tata Memorial Centre, Navi Mumbai, India - 410210

2 Molecular Functional Imaging Lab, Advanced Centre for Treatment, Research and Education in Cancer, Tata Memorial Centre, Navi Mumbai, India - 410210

3 Homi Bhabha National Institute, Anushakti Nagar, Mumbai, India - 400094

4 Laboratory of Medicinal Chemistry and Cell Biology, Department of Chemical Sciences, Tata Institute of Fundamental Research, Mumbai, India - 400005

5 Department of Pathology, Tata Memorial Hospital, Mumbai, India – 400012

6 Indian Institute of Science Education and Research, Bhopal, India – 462066

7 Indian Institute of Science Education and Research, Kolkata, India – 741246

*** Corresponding author, Email ID: pray@actrec.gov.in**

Supplementary Figure 1

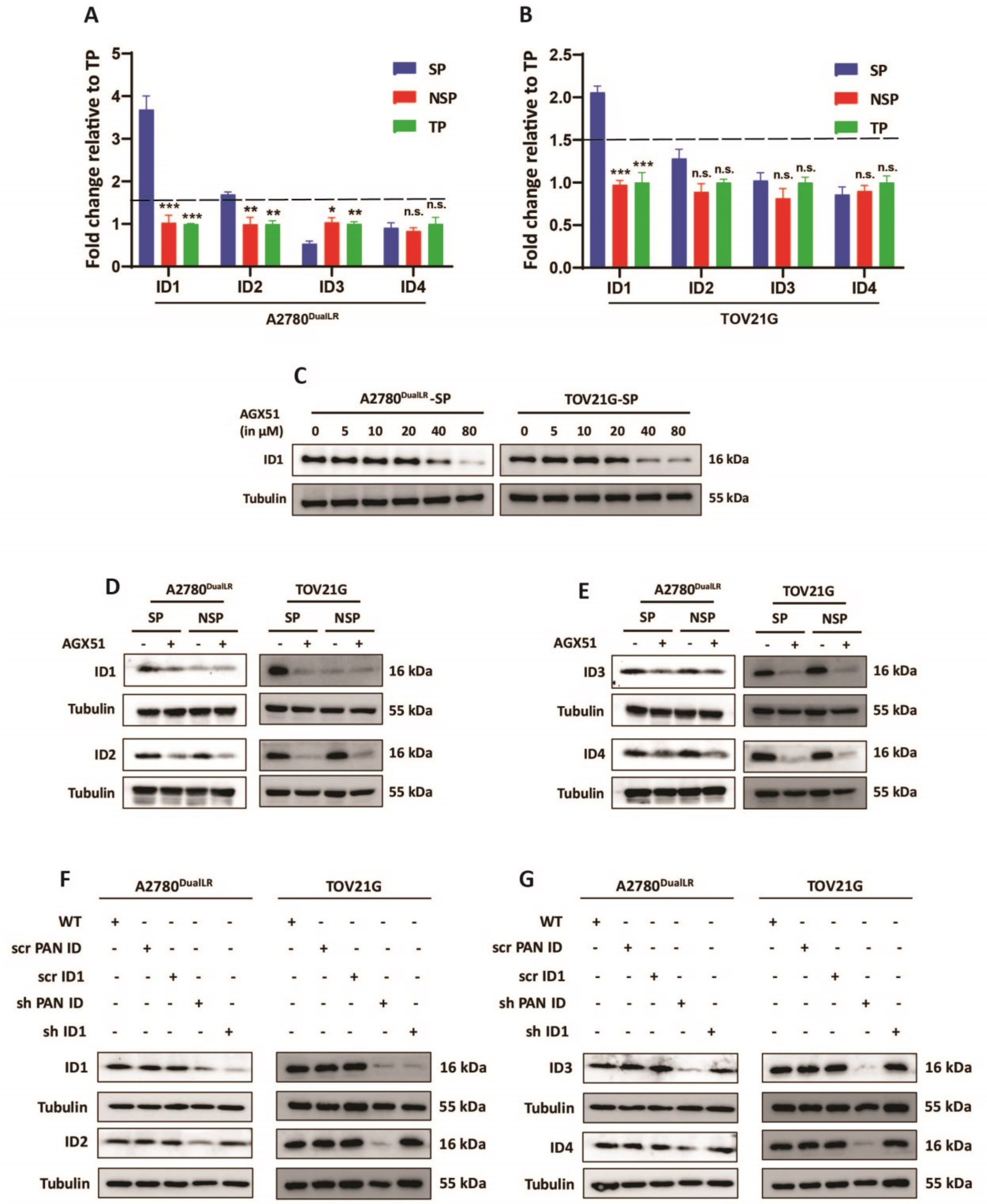

Figure S1: (A, B) RT-qPCR profiling for ID genes (ID1-ID4) revealed that ID1 had the highest upregulation (fold change above 1.5 threshold relative to TP cells) in SP cells (3.6-fold and 2-fold in A2780^{DualLR}-SP and TOV21G-SP respectively) compared to NSP and TP cells. ID2 was

1.7-fold upregulated in SP cells compared to NSP and TP of A2780^{DualLR} cells but non-significantly increased in SP cells compared to NSP and TP of TOV21G. There were no significant changes in the expression of ID3 and ID4 across SP, NSP and TP of A2780^{DualLR} and TOV21G cells. Data represented as mean ± SEM, *p<0.05, **p<0.01, ***p<0.001, n.s.-non-significant. **(C)** A2780^{DualLR} and TOV21G cells were treated with increasing concentrations of AGX51 (0, 5, 10, 20, 40, and 90 µM) for 24 hours. Protein levels of ID1 were analysed by Western blot. 40 µM AGX51 was identified as the optimal concentration for ID1 degradation in both cell lines. **(D, E)** SP and NSP cells were treated with 40 µM AGX51 for 24 hours. Protein levels of ID1, ID2, ID3, and ID4 were analysed by Western blot. AGX51 treatment effectively reduced the expression of all four ID proteins in both SP and NSP cells of A2780^{DualLR} and TOV21G cells. **(F, G)** Western blot analysis was performed to assess the expression levels of ID1, ID2, ID3, and ID4 proteins in A2780^{DualLR} and TOV21G cells following shRNA-mediated knockdown. Cells were transduced with sh PAN-ID (targeting all ID proteins), sh ID1 (targeting only ID1), or their respective scrambled controls (scr PAN-ID and scrID1). Wild-type (WT) non-transduced cells served as a baseline control. sh PAN-ID effectively downregulated all four ID proteins, while sh ID1 specifically reduced ID1 levels. The scrambled controls showed no significant changes in ID protein expression compared to WT cells in both cell lines. SP: Side Population, NSP: Non-Side Population, TP: Total Population,

Supplementary Figure 2

A

Cisplatin (in ng/uL)	A2780^{DualLR} WT								
	Replicate 1			Replicate 2			Replicate 3		
0	100.000	100.000	100.000	100.000	100.000	100.000	100.000	100.000	100.000
250	90.814	86.813	88.439	78.174	94.454	92.382	84.964	85.593	82.742
500	86.013	73.711	81.255	75.519	86.915	78.363	83.294	71.186	78.251
1000	86.117	68.893	69.034	75.685	79.289	76.013	76.213	68.182	72.734
2000	53.758	48.436	46.821	47.552	51.646	45.138	54.733	42.450	42.947

Cisplatin (in ng/uL)	A2780^{DualLR} shPAN ID								
	Replicate 1			Replicate 2			Replicate 3		
0	100.000	100.000	100.000	100.000	100.000	100.000	100.000	100.000	100.000
250	51.027	65.106	51.383	38.769	61.751	48.980	52.273	69.112	51.799
500	38.014	57.447	36.759	31.692	51.152	46.531	40.152	40.541	43.885
1000	26.027	29.362	26.087	17.538	27.650	22.449	32.197	20.077	23.381
2000	16.438	20.426	19.368	12.615	18.894	16.735	17.045	16.988	15.468

Cisplatin (in ng/uL)	A2780^{DualLR} shID1								
	Replicate 1			Replicate 2			Replicate 3		
0	100.000	100.000	100.000	100.000	100.000	100.000	100.000	100.000	100.000
250	57.525	49.837	61.765	45.329	51.481	44.660	51.333	62.500	58.007
500	33.445	36.156	44.853	43.253	37.037	37.217	42.667	46.970	47.687
1000	22.074	19.218	27.574	23.183	26.667	21.359	20.667	25.379	26.335
2000	13.712	17.264	15.441	14.879	16.667	17.476	16.333	20.076	17.438

B

Cisplatin (in ng/uL)	TOV21G WT								
	Replicate 1			Replicate 2			Replicate 3		
0	100.000	100.000	100.000	100.000	100.000	100.000	100.000	100.000	100.000
250	90.728	89.423	92.033	76.202	76.690	77.907	76.701	79.767	75.000
500	81.185	88.304	86.813	74.279	77.855	66.512	63.299	73.488	71.272
1000	91.289	81.287	79.121	73.558	51.049	60.698	76.082	66.047	45.614
2000	25.087	23.099	22.802	19.231	20.746	20.233	16.289	16.279	17.105

Cisplatin (in ng/uL)	TOV21G shPAN ID								
	Replicate 1			Replicate 2			Replicate 3		
0	100.000	100.000	100.000	100.000	100.000	100.000	100.000	100.000	100.000
250	87.308	52.977	67.584	50.182	59.155	50.548	59.383	90.196	68.994
500	41.562	27.582	32.416	49.453	40.205	37.397	49.630	49.321	38.225
1000	11.855	10.571	12.891	11.179	8.707	10.411	12.469	16.440	11.361
2000	3.905	3.159	3.630	3.159	3.457	3.699	3.086	4.525	3.077

Cisplatin (in ng/uL)	TOV21G shID1								
	Replicate 1			Replicate 2			Replicate 3		
0	100.000	100.000	100.000	100.000	100.000	100.000	100.000	100.000	100.000
250	85.471	68.733	85.596	71.791	67.459	60.235	75.205	83.153	105.992
500	30.890	28.706	22.022	24.465	38.548	22.588	32.877	40.804	28.629
1000	7.984	6.065	6.233	6.952	6.508	5.882	5.890	7.573	6.791
2000	3.927	3.774	4.155	3.610	3.129	3.412	3.699	4.173	3.728

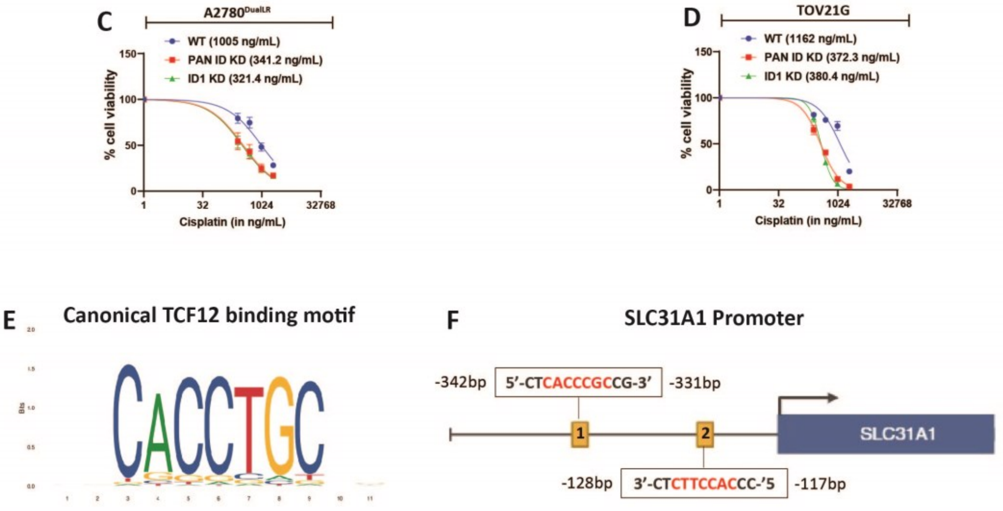

Figure S2: (A, B) Raw MTT assay data for A2780^{DualLR} and TOV21G cells treated with increasing concentrations of cisplatin. **(C, D)** Dose-response curves showing cell viability of wild-type (WT), shPAN-ID, and shID1 transduced A2780^{DualLR} and TOV21G cells in response to cisplatin. shRNA-mediated knockdown of PAN-ID or ID1 resulted in a marked decrease in cell viability and a 3-fold reduction in IC50 values compared to WT cells. IC50 values: A2780^{DualL}R (WT: 1005 ng/mL; shPAN-ID: 341.2 ng/mL; shID1: 321.4 ng/mL) and TOV21G (WT: 1162 ng/mL; shPAN-ID: 372.3 ng/mL; shID1: 380.4 ng/mL). Data represent mean ± SEM, n=3. **(E)** Schematic of human TCF12 canonical TCF12 binding motif obtained from JASPER Database. **(F)** Schematic of the SLC31A1 promoter showing two high-scoring putative TCF12 binding sites predicted by JASPAR.

Supplementary Figure 3

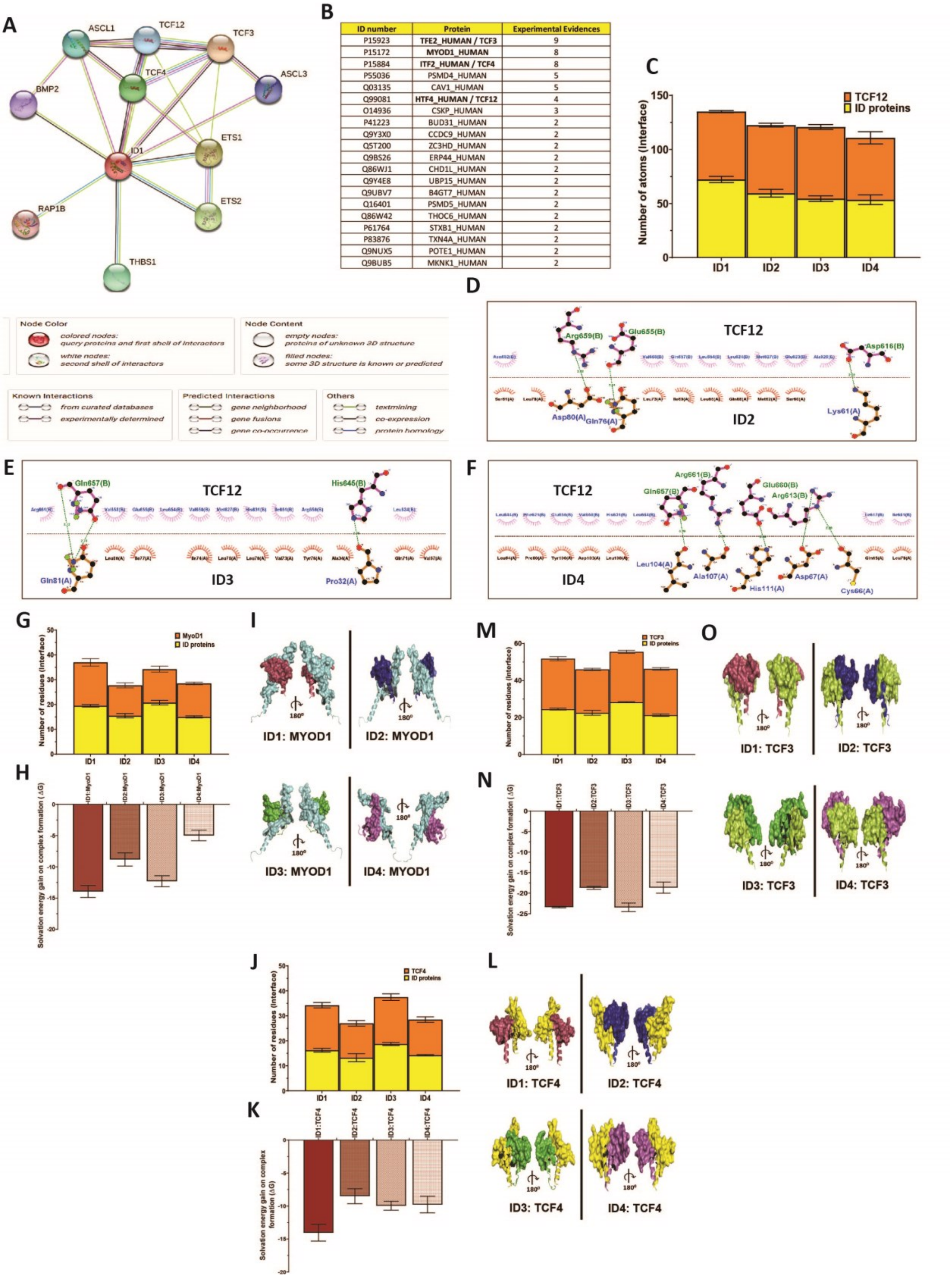

ID number	Protein	Experimental Evidences
P15923	**TFE2_HUMAN / TCF3**	9
P15172	**MYOD1_HUMAN**	8
P15884	**ITF2_HUMAN / TCF4**	8
P55036	PSMD4_HUMAN	5
Q03135	CAV1_HUMAN	5
Q99081	**HTF4_HUMAN / TCF12**	4
O14936	CSKP_HUMAN	3
P41223	BUD31_HUMAN	2
Q9Y3X0	CCDC9_HUMAN	2
Q5T200	ZC3HD_HUMAN	2
Q9BS26	ERP44_HUMAN	2
Q86WJ1	CHD1L_HUMAN	2
Q9Y4E8	UBP15_HUMAN	2
Q9UBV7	B4GT7_HUMAN	2
Q16401	PSMD5_HUMAN	2
Q86W42	THOC6_HUMAN	2
P61764	STXB1_HUMAN	2
P83876	TXN4A_HUMAN	2
Q9NUX5	POTE1_HUMAN	2
Q9BUB5	MKNK1_HUMAN	2

Figure S3: (A, B) STRING and AGILE analysis showing the potential interactions and functional relevance of ID1 **(C)** Graph showing the number of atoms involved in stabilizing the ID1-4 interaction with TCF12. **(D-F)** Lig-plot analysis of **(D)** ID2:TCF12, **(E)** ID3:TCF12 and **(F)** ID4:TCF12 dimer revealing dramatic decrease in H-bonds and increase in the van-der waal interaction. **(G, J, N)** Graph showing the total number of residues involved in stabilizing the **(G)** ID1-4: MyoD1 dimer, **(J)** ID1-4: TCF4 dimer and **(M)** ID1-4: TCF3 dimer. Each bar denotes the mean ± S.D. of top four HADDOCK structures. **(H, K, N)** Graph showing the thermodynamic feasibility of **(H)** ID1-4 interaction with MyoD1, **(K)** ID1-4 interaction with TCF4 and **(N)** ID1-4 interaction with TCF3. **(I, L, O)** Representative 3-D structures of ID1-4 interactions with **(I)** MyoD1 **(L)** TCF4 and **(O)** TCF3.

Supplementary Figure 4

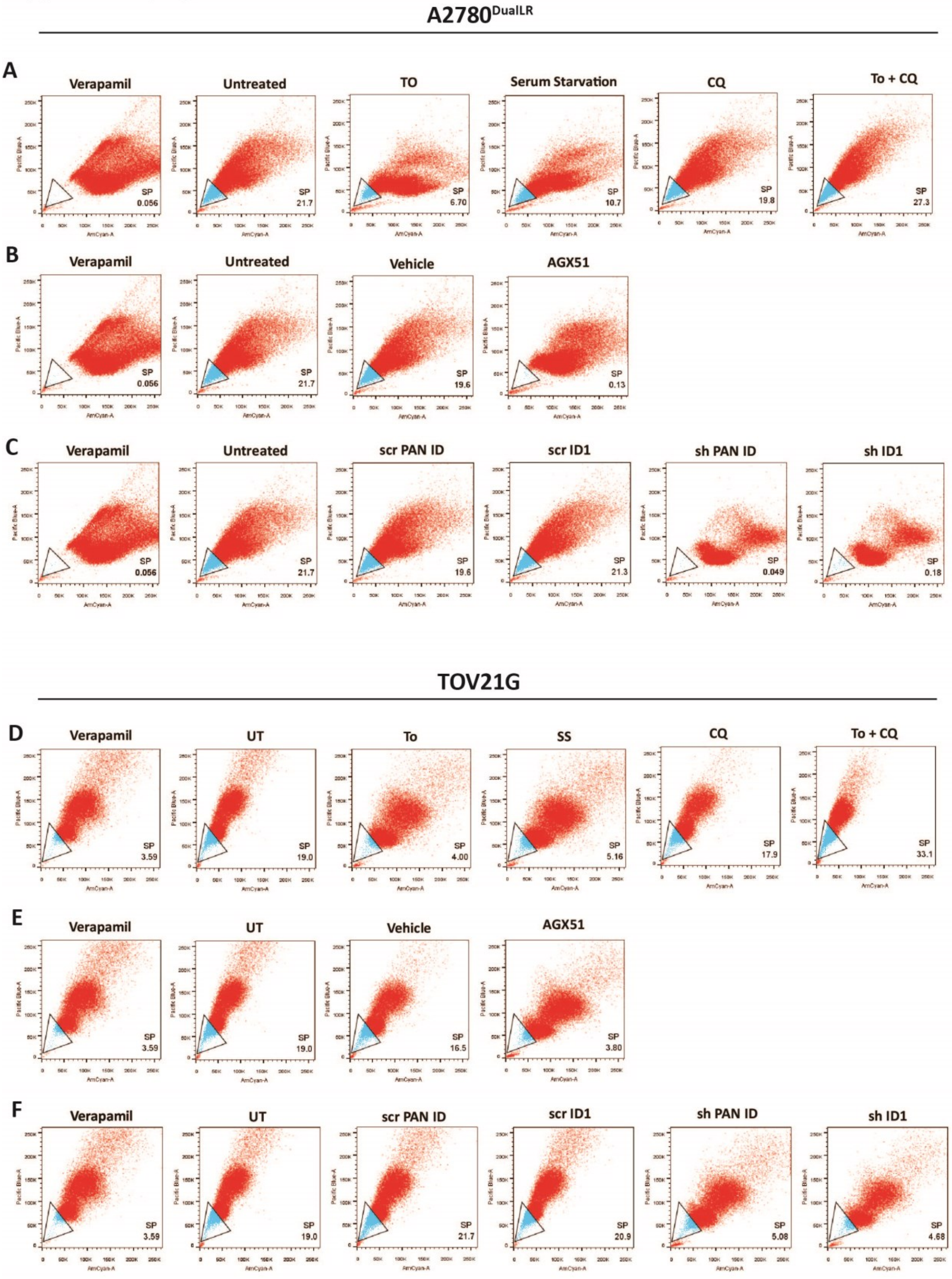

Figure S4: (A, D) FACS plots showing the percentage of SP cells after autophagy modulation in A2780^{DualLR} (A) and TOV21G (D) cells. Treatments include autophagy induction (Torin 1 and serum starvation), blockade (Chloroquine, CQ), and dual manipulation (Torin 1 + CQ). **(B, E)** FACS plots displaying the percentage of SP cells following AGX51 treatment (pharmacological depletion of ID1) in A2780^{DualLR} (B) and TOV21G (E) cells. Vehicle control showed no significant change compared to untreated cells. **(C, F)** FACS plots illustrating the percentage of SP cells after genetic knockdown (shPAN-ID and shID1) in A2780^{DualLR} (C) and TOV21G (F) cells. Scrambled controls (scrPAN-ID and scrID1) showed no significant changes compared to non-transduced wild-type cells. SP cells were identified based on DCV dye efflux. Data represent n=3 independent experiments. The experiments were conducted together as single set of data: (Set A-C) and (Set D-F). For each set a single Verapamil-treated control and a single untreated has been control utilized for comparative analysis. SP: Side Population, NSP: Non-Side Population, TP: Total Population, TO: Torin 1, CQ: Chloroquine, AGX51: PAN ID Inhibitor, SS: Serum Starvation, scr PAN-ID: Scrambled PAN-ID, scr ID1: scrambled ID1, sh PAN ID: PAN ID Knockdown, sh ID1: ID1 knockdown

Supplementary Figure 5

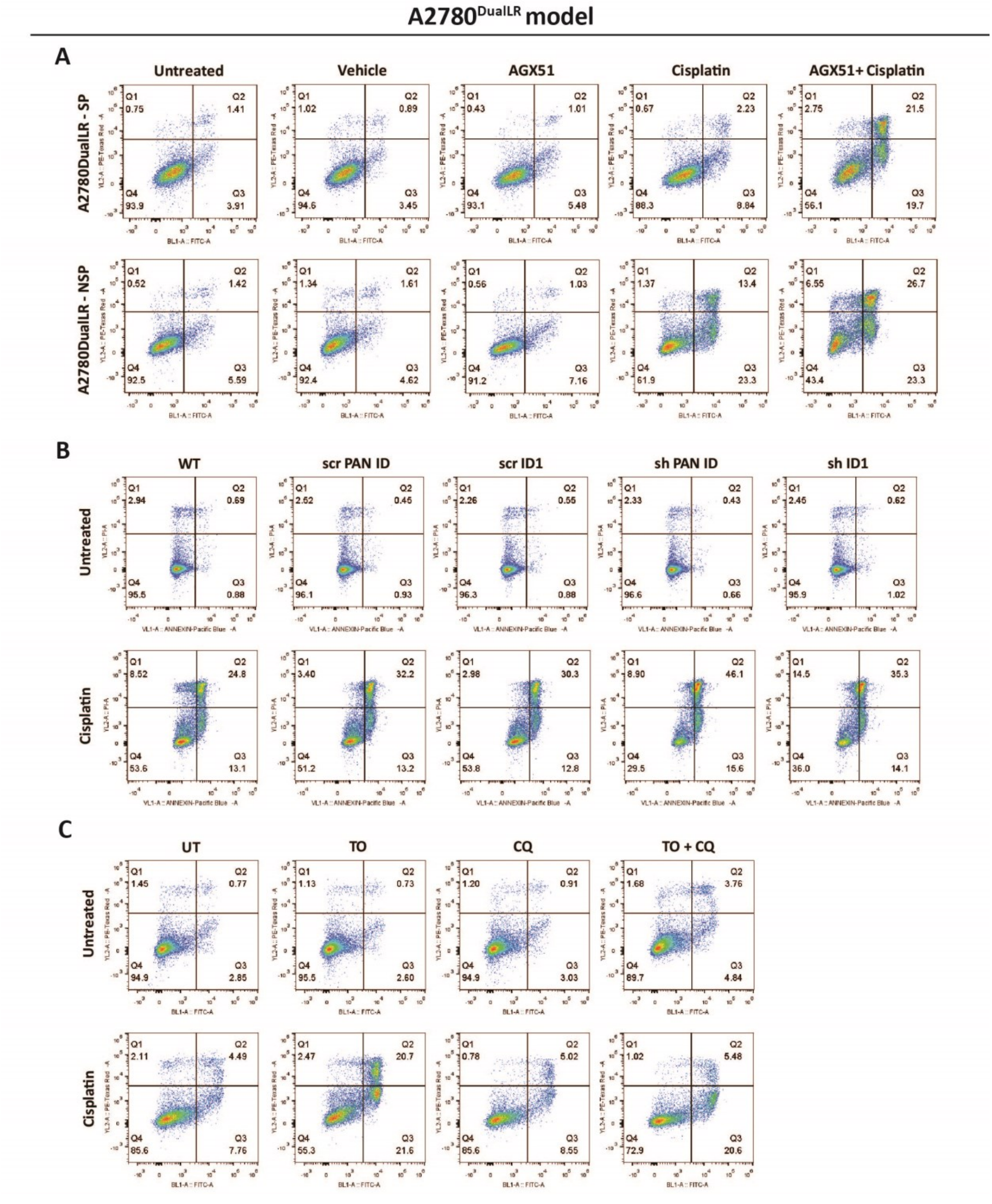

Figure S5: Annexin V-PI FACS plots showing the percentage of cell death (necrosis + early and late apoptosis) after cisplatin treatment in A2780^{DualLR} (SP, NSP and TP cells) under various conditions: **(A)** Pharmacological ID1 depletion using AGX51 in SP and NSP cells: The

combination of AGX51 and cisplatin showed a synergistic effect in both cell populations. NSP cells showed more death compared to SP indicating their resistant nature. **(B)** Genetic knockdown of ID1. shPAN-ID and shID1 cells exhibited increased cell death compared to their respective scrambled controls (scr PAN-ID and scr ID1). Scrambled controls showed no significant difference in cell death compared to wild-type (WT) non-transduced cells. **(C)** ID1 depletion via autophagy modulation: Torin 1 treatment combined with cisplatin led to increased cell death. This effect was partially reversed in the Torin 1 + Chloroquine (TO+CQ) group treated with cisplatin, suggesting that the observed effects are mediated by autophagy induction. Cells were treated with 10X IC50 of cisplatin of A2780^{DualLR} for 24 hours. SP: Side Population, NSP: Non-Side Population, TP: Total Population, TO: Torin 1, CQ: Chloroquine, AGX51: PAN ID Inhibitor, SS: Serum Starvation, scr PAN-ID: Scrambled PAN-ID, scr ID1: scrambled ID1, sh PAN ID: PAN ID Knockdown, sh ID1: ID1 knockdown

Supplementary Figure 6

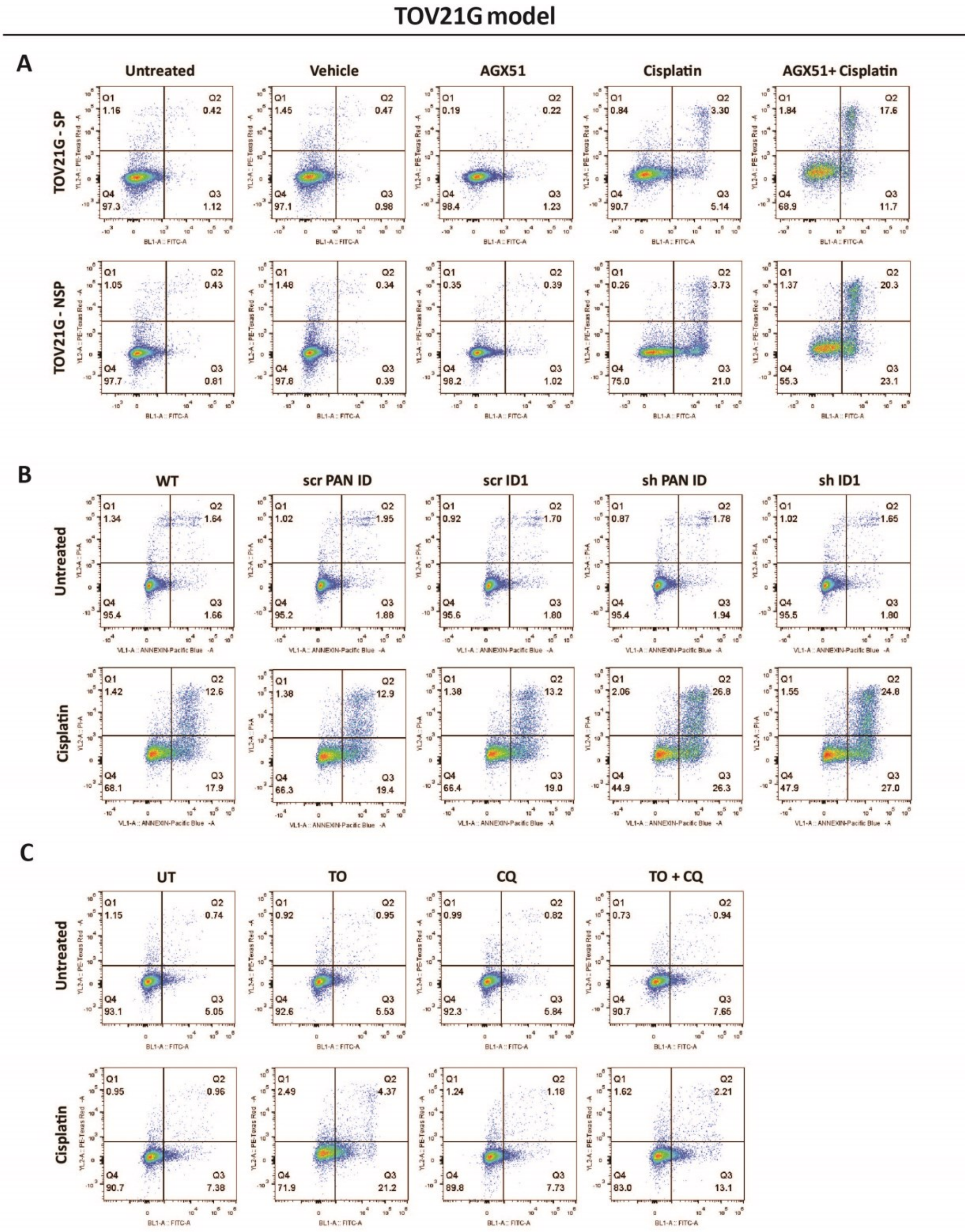

Figure S6: Annexin V-PI FACS plots showing the percentage of cell death (necrosis + early and late apoptosis) after cisplatin treatment in TOV21G (SP, NSP and TP cells) under various conditions: **(A)** Pharmacological ID1 depletion using AGX51 in SP and NSP cells: The combination of AGX51 and cisplatin showed a synergistic effect in both cell populations. NSP

cells showed more death compared to SP indicating their resistant nature. **(B)** Genetic knockdown of ID1. shPAN-ID and shID1 cells exhibited increased cell death compared to their respective scrambled controls (scr PAN-ID and scr ID1). Scrambled controls showed no significant difference in cell death compared to wild-type (WT) non-transduced cells. **(C)** ID1 depletion via autophagy modulation: Torin 1 treatment combined with cisplatin led to increased cell death. This effect was partially reversed in the Torin 1 + Chloroquine (TO+CQ) group treated with cisplatin, suggesting that the observed effects are mediated by autophagy induction. Cells were treated with 10X IC50 of cisplatin of TOV21G for 24 hours. SP: Side Population, NSP: Non-Side Population, TP: Total Population, TO: Torin 1, CQ: Chloroquine, AGX51: PAN ID Inhibitor, SS: Serum Starvation, scr PAN-ID: Scrambled PAN-ID, scr ID1: scrambled ID1, sh PAN ID: PAN ID Knockdown, sh ID1: ID1 knockdown

Supplementary Figure 7

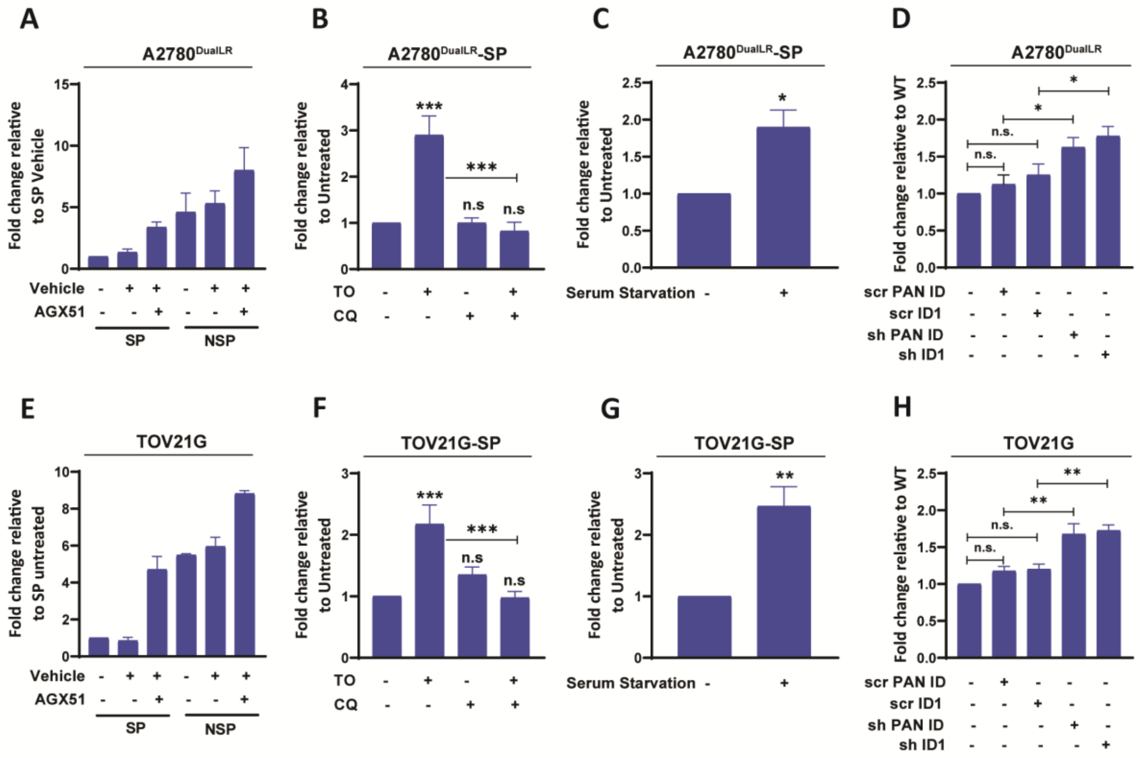

Figure S7: (A, E) Densitometric analysis of SLC31A1 protein levels in A2780^{DualLR} and TOV21G cells (n=2). SP cells showed lower SLC31A1 expression compared to NSP and TP cells. ID1 depletion by AGX51 treatment increased SLC31A1 levels in both SP and NSP cells, with the highest increase observed in NSP cells. Vehicle control (0.4% DMSO) showed no changes in protein levels. **(B, F)** Quantification of SLC31A1 protein levels following autophagy-mediated depletion of ID1 (n=3). Torin1 treatment led to significant upregulation of SLC31A1 protein. Simultaneous treatment with Torin1 and CQ resulted in decreased SLC31A1 protein levels compared to Torin1 alone in both A2780^{DualLR}-SP and TOV21G-SP cells, indicating autophagy induction-specific effects. **(C, G)** Densitometric analysis of SLC31A1 protein levels after serum starvation (n=3). Both A2780^{DualLR}-SP and TOV21G-SP cells showed significant increases in SLC31A1 protein levels following serum starvation. **(D, H)** Quantification of SLC31A1 protein levels following genetic depletion of ID1 (n=3). Both sh PAN-ID and sh ID1 depletion of ID1 led to increased levels of SLC31A1 protein, with the

highest increase observed in shID1 cells of both A2780^{DualLR} and TOV21G cells. Scrambled controls (scr PAN ID and scr ID1) showed no significant changes in SLC31A1 protein levels compared to non-transduced wild-type cells. Data represents mean ± SEM. Statistical analysis was performed using Student's t-test for comparing untreated and drug-treated group. For comparisons involving multiple groups, one-way ANOVA was used, followed by Dunnett's post-hoc test when comparing multiple groups to a control, or Bonferroni post-hoc test when comparing all groups to each other, whichever is appropriate. *$p<0.05$, **$p<0.01$, ***$p<0.001$, n.s.- non-significant. SP: Side Population, NSP: Non-Side Population, TP: Total Population, TO: Torin 1, CQ: Chloroquine, AGX51: PAN ID Inhibitor, SS: Serum Starvation, scr PAN-ID: Scrambled PAN-ID, scr ID1: scrambled ID1, sh PAN ID: PAN ID Knockdown, sh ID1: ID1 knockdown

Table S1. List of antibodies used in this study

**Primary Antibodies**			
**Antibody**	**Source**	**Identifier**	**Company**
ABCG2	Rabbit	A17908	ABclonal, MA, USA
ID1	Mouse	sc-133104	SCBT, TX, USA
ID2	Mouse	sc-398104	SCBT, TX, USA
ID3	Mouse	sc-56712	SCBT, TX, USA
ID4	Mouse	sc-365656	SCBT, TX, USA
LAMP1	Mouse	H4A3	DSHB, IA, USA
LC3B	Rabbit	2775S	CST, MA, USA
NANOG	Mouse	sc-293121	SCBT, TX, USA
OCT-3/4	Rat	MAB1759	Biotechne, MN, USA
p62/SQSTM1	Rabbit	ab155686	CST, MA, USA
SLC31A1	Rabbit	A0773	ABclonal, MA, USA
SLC31A1 (I.H.C. grade)	Rabbit	APREST72883	Sigma Aldrich, DA, DE
TCF12	Rabbit	A4146	ABclonal, MA, USA
TCF12 (ChIP grade)	Mouse	sc-28364-X	SCBT, TX, USA
TCF12	Rabbit	11825	CST, MA, USA
α-Tubulin	Mouse	T5168	Sigma Aldrich, DA, DE
Rabbit Gamma Globulin Control	Rabbit	31887	Invitrogen, USA
Mouse Gamma Globulin Control	Mouse	31878	Invitrogen, USA
**Secondary antibodies**			
Alexa Fluor™ 568	Rat	A11004	Thermofisher, MA USA
Anti-Mouse IgG HRP conjugate	Goat	4416	Thermofisher, MA USA

Anti-Rabbit IgG HRP conjugate	Goat	A6154	Thermofisher, MA USA
DyLight™ 488	Rabbit	35552	Thermofisher, MA USA
DyLight™ 633	Mouse	35512	Thermofisher, MA USA

Table S2. List of oligonucleotide sequences used in this study

**Primer Name**	**Sequences**	**Purpose**
shPAN-ID	5'-CTACGACATGAACGGCTGTTA-3'	PAN ID shRNA plasmid construction
shID1	5'-AGGTGAGCAAGGTGGAGATTC-3'	ID1 shRNA plasmid construction
scr PANID	5'- ACTTCGAGAACTGTCGGAACT-3'	scrambled PAN ID shRNA plasmid construction
scrID1	5'-GGAGTGTGCGTAGACGATAAG-3'	scrambled ID1 shRNA plasmid construction
SLC31A1 F	5'-AGGACTCAAGATAGCCCGAGAGA-3'	qRT-PCR
SLC31A1 R	5'-CCTGGGACAGGCATGGAA-3'	qRT-PCR
OCT4A F	5'- CCTGAAGCAGAAGAGGATCACC-3'	qRT-PCR
OCT4A R	5'-AAAGCGGCAGATGGTCGTTTGG-3'	qRT-PCR
NANOG F	5'-CTCCAACATCCTGAACCTCAGC-3'	qRT-PCR
NANOG R	5'-CGTCACACCATTGCTATTCTTCG-3'	qRT-PCR
GAPDH F	5'-TGCACCACCAACTGCTTAGC-3'	qRT-PCR
GAPDH R	5'-GGCATGGACTGTGGTCATGAG-3'	qRT-PCR
Site 1 F	5'- AACAATCCGCCGGTATCTCT-3'	CHIP-qRT-PCR

Site 1 R	5’- TGAAAGCCAACGCAATGTTCG-3’	CHIP-qRT-PCR
Site 2 F	5’- ACGGTCTCTGGACCGAAAGT-3’	CHIP-qRT-PCR
Site 2 R	5’-CGCGCGAGCCTCTCATTT-3’	CHIP-qRT-PCR
GAPDH F	5’-TACTAGCGGTTTTACGGGCG-3’	CHIP-qRT-PCR
GAPDH R	5’-TCGAACAGGAGGAGCAGAGAGCGA-3’	CHIP-qRT-PCR
